# Exogenous glucocorticoids to improve extinction learning for post-traumatic stress disorder patients with hypothalamic–pituitary–adrenal-axis dysregulation: a study protocol description

**DOI:** 10.1080/20008066.2024.2364441

**Published:** 2024-07-08

**Authors:** Laura de Nooij, Lisa Wirz, Emma Heling, Mariana Pais, Gert-Jan Hendriks, Robbert-Jan Verkes, Benno Roozendaal, Erno J. Hermans

**Affiliations:** a Department of Cognitive Neuroscience, Radboud University Medical Center, Donders Institute for Brain, Cognition and Behaviour, Nijmegen, The Netherlands; b Cognitive Psychology, Ruhr-University Bochum, Bochum, Germany; c Behavioural Science Institute, Radboud University, Nijmegen, The Netherlands; d ‘Overwaal’ Center of Expertise for Anxiety, Obsessive Compulsive and Posttraumatic Stress Disorders, Institution for Integrated Mental Health Care “Pro Persona”, Nijmegen, The Netherlands; e Kairos Forensic Care, Pompestichting, Nijmegen, The Netherlands

**Keywords:** PTSD, early-life stress, HPA axis, glucocorticoids, extinction learning, emotional memory, TEPT, estrés en la vida temprana, eje HHA, glucocorticoides, aprendizaje de extinción, recuerdo emocional

## Abstract

**Background:** Trauma-focused treatments for post-traumatic stress disorder (PTSD) are effective for many patients. However, relapse may occur when acquired extinction memories fail to generalize beyond treatment contexts. A subgroup of PTSD patients – potentially with substantial exposure to early-life adversity (ELA) – show dysregulation of the hypothalamic–pituitary–adrenal (HPA) axis, which results in lower cortisol levels. Glucocorticoids, including cortisol, appear to facilitate strength and generalization of emotional memories.

**Objective:** We describe the protocol of an integrated PTSD study. We investigate (A) associations between HPA-axis dysregulation, ELA, epigenetic markers, and PTSD treatment outcome (observational study); and (B) effects of exogenous glucocorticoids on strength and generalization of extinction memories and associated neural mechanisms [pharmacological intervention study with functional magnetic resonance imaging (fMRI)]. The objective is to provide proof of concept that PTSD patients with HPA-axis dysregulation often experienced ELA and may show improved strength and generalization of extinction learning after glucocorticoid administration.

**Method:** The observational study (*n *= 160 PTSD group, *n *= 30 control group) assesses ELA, follow-up PTSD symptoms, epigenetic markers, and HPA-axis characteristics (salivary cortisol levels during low-dose dexamethasone suppression test and socially evaluated cold-pressor test). The pharmacological intervention study (*n *= 80 PTSD group, with and without HPA-axis dysregulation) is a placebo-controlled fMRI study with a crossover design. To investigate strength and generalization of extinction memories, we use a differential fear acquisition, extinction, and extinction recall task with spatial contexts within a virtual environment. Prior to extinction learning, 20 mg hydrocortisone or placebo is administered. During next-day recall, strength of the extinction memory is determined by recovery of skin conductance and pupil dilation differential responding, whereas generalization is assessed by comparing responses between different spatial contexts.

**Conclusion:** The integrated study described in the current protocol paper could inform a personalized treatment approach in which these PTSD patients may receive glucocorticoids as a treatment enhancer in trauma-focused therapies.

**Trial registration:** The research project is registered in the European Union Drug Regulating Authorities Clinical Trials (EudraCT) database, https://eudract.ema.europa.eu/, EudraCT number 2020-000712-30.

## HIGHLIGHTS

- This protocol reports a proof-of-concept study for glucocorticoids as an enhancer for PTSD treatment.
- The study examines whether glucocorticoids enhance the strength and generalization of extinction memory.
- A further aim is to identify HPA-axis-related PTSD subgroups that may particularly benefit.

## Abstract (es)

**Antecedentes:** Los tratamientos centrados en trauma para el trastorno de estrés postraumático (TEPT) son efectivos para muchos pacientes. Sin embargo, las recaídas pueden ocurrir cuando la extinción de los recuerdos adquiridos no logra generalizarse más allá de los contextos del tratamiento. Un subgrupo de pacientes con TEPT, potencialmente con una exposición importante a adversidades tempranas en la vida (ELA por sus siglas en inglés), muestran desregulación del eje hipotálamo-hipófisis-adrenal (HHA), lo cual resulta en niveles de cortisol más bajos. Los glucocorticoides, incluyendo el cortisol, parece facilitar la fuerza y generalización de los recuerdos emocionales.

**Objetivo:** Aquí describimos el protocolo de un estudio de TEPT integrado. Investigamos (A) asociaciones entre la desregulación del eje HHA, ELA, marcadores epigenéticos y resultados del tratamiento del TEPT (estudio observacional); y (B) los efectos de los glucocorticoides exógenos sobre la fuerza y generalización de la extinción de los recuerdos y los mecanismos neurales asociados (estudio de intervenciones farmacológicas con imágenes de resonancia magnética funcional [fMRI]). El objetivo es proporcionar prueba de concepto de que los pacientes con TEPT con desregulación del eje HHA con frecuencia han experimentado ELA y pueden mostrar una mayor fuerza y generalización del aprendizaje de extinción tras la administración de glucocorticoides.

**Métodos:** El estudio observacional (*n*=160 grupo TEPT, *n*=30 grupo control) evalúa ELA, seguimiento de los síntomas de TEPT, marcadores epigenéticos y características del eje HHA (niveles de cortisol en saliva durante el test de supresión de dexametasona en bajas dosis y la prueba de estimulación por frio evaluada socialmente). El estudio de intervención farmacológica (*n*=80 grupo TEPT, con y sin desregulación del eje HHA) es un estudio de fMRI controlado con placebo con diseño cruzado. Para investigar la fuerza y generalización de la extinción de los recuerdos, utilizamos una tarea de adquisición del miedo, extinción y recuerdo de extinción diferencial con contextos espaciales dentro de un entorno virtual. Previo al aprendizaje de extinción, se administra 20 mg. de hidrocortisona o placebo. Durante el recuerdo del siguiente día, la fuerza de la extinción del recuerdo está determinada por la recuperación de la conductancia táctil y la respuesta diferencial de la dilatación pupilar, mientras que la generalización se evalúa comparando respuestas entre diferentes contextos espaciales.

**Conclusión:** El estudio integrado descrito en el artículo del protocolo actual podría informar de una aproximación de tratamiento personalizado en el que los pacientes con TEPT podrían recibir glucocorticoides como potenciador del tratamiento en terapia centradas en trauma.

## 1. Introduction

Post-traumatic stress disorder (PTSD) is a common and debilitating psychiatric disorder that may develop after experiencing or witnessing one or more severely distressing and life-threatening events (American Psychiatric Association, 2013). The disorder is characterized by persistent and intrusive flashbacks, recurring nightmares or upsetting memories, avoidance, negative feelings, and changes in arousal and reactivity (e.g. difficulty concentrating, sleep problems, irritability, and/or hypervigilance) (American Psychiatric Association, 2013). First-line treatments for PTSD are trauma-focused psychotherapies, for example, prolonged exposure and eye movement desensitization reprocessing (Rousseau et al., 2019).

An important common factor of these treatments is that they are based on the extinction of conditioned fear by re-exposure to trauma-related memories. During exposure therapy (i.e. all forms of exposure-based trauma-focused treatment), patients are exposed to fear-provoking situations and activities in order to promote extinction of the fear response as well as learning of safety signals. This involves emotional learning, since new and adaptive safety memories are created that compete with maladaptive aversive memories (Ressler & Mayberg, 2007). If successful, recall of safety memories prevents the expression of traumatic memories. Exposure therapy is evidence based and generally effective for PTSD (Bradley et al., 2005; Cusack et al., 2016; Ehring et al., 2014; Hoffman et al., 2018), but not all patients achieve complete remission with these treatments. Failure to inhibit excessive fear and the inability to learn safety are hallmarks of PTSD and other fear-related disorders and contribute to non-response (Schottenbauer et al., 2014; Wessa & Flor, 2007; Wicking et al., 2016). In addition, return of fear is another well-recognized problem (Bandelow et al., 2007; Bouton et al., 2012). Exposure therapy is therefore insufficiently effective in the long term for around 30–50% of PTSD patients (Bradley et al., 2005; Cusack et al., 2016). This indicates the need to investigate innovative interventions that may enhance treatment success in non-responder and relapsing subgroups of PTSD patients, paving the way for a personalized treatment approach.

However, delineation of PTSD subgroups into meaningful subgroups that can provide information about treatment trajectories remains a challenge. Individuals with ‘complex PTSD’ can experience a more chronic course that often links back to substantial exposure to early-life adversity (ELA), for instance, chronic abuse or neglect (World Health Organization, 2018). In accordance with clinical observations, some previous studies suggests that individuals with ELA more often show less favourable PTSD treatment outcome (Bosch et al., 2020; Dorrepaal et al., 2014; Hoeboer et al., 2021). Other studies indicate that this subgroup generally responds well, albeit with lower effect size (Ehring et al., 2014; Wagenmans et al., 2018). Here, individual differences in treatment outcome may be more directly related to the adaptation of certain biological systems in response to chronic ELA. Previous research suggests that ELA may play an important role with regard to hypothalamic–pituitary–adrenal (HPA)-axis dysregulation in PTSD (Houtepen et al., 2016; Pitman et al., 2012; Zoladz & Diamond, 2013). Altered HPA-axis regulation could consequently affect the development of brain structure and functioning (Raymond et al., 2018). Neurobiological variation in fear-regulatory neural circuits that affect emotional learning may predict individual differences in exposure therapy treatment outcome. These factors may provide stress- and fear-related biological markers that could identify non-responder and relapsing individuals with PTSD and corresponding targets to develop more personalized PTSD treatments.

In PTSD, enhanced HPA-axis feedback regulation can lead to exaggerated suppression of cortisol, which results in lower circulating cortisol levels (Goenjian et al., 1996; Grossman et al., 2003; Pitman et al., 2012; Stein et al., 1997; Yehuda, 2009; Yehuda et al., 1993, 1995, 2002, 2004). Although most meta-analyses support this notion of HPA-axis dysregulation (Meewisse et al., 2007; Morris et al., 2012; Pan et al., 2018), another meta-analysis (Klaassens et al., 2012) and multiple studies that failed to replicate these findings suggest that low cortisol may only be observed under certain conditions, perhaps only within subgroups of individuals with PTSD (Meewisse et al., 2007; Pitman et al., 2012; Zoladz & Diamond, 2013). DNA methylation can suppress gene transcription and, as such, contribute to individual differences in glucocorticoid signalling (Turecki & Meaney, 2016; Zhang et al., 2013). Previous research indicated that decreased methylation of the glucocorticoid receptor (GR) gene *NR3C1* is associated with ELA (Oberlander et al., 2008; Parent et al., 2018; Weaver et al., 2007). Moreover, allele-specific DNA demethylation of *FKBP5* (a co-chaperone that regulates GR activity) mediates gene-by-ELA interactions and induces long-term HPA-axis dysregulation (Klengel et al., 2013; Koenig et al., 2018; Mehta et al., 2011). HPA-axis dysregulation and methylation status of *NR3C1* and *FKBP5* are also associated with increased lifetime risk for PTSD development if exposed to traumatic events (Labonté et al., 2014; van Zuiden et al., 2011; Vukojevic et al., 2014; Yehuda et al., 2015b). Beyond these observational findings, experimental research with rodents has provided causal support that ELA can lead to HPA-axis dysregulation. ELA was shown to cause lower glucocorticoid levels, which was accompanied by an upregulation of GRs within the hippocampus and medial prefrontal cortex and decreased later-life social behaviour, of which the latter was reinstated with glucocorticoid administration (Perry et al., 2019). Gene expression patterns thus tune the sensitivity of the stress response and negative feedback inhibition via epigenetic mechanisms.

Dysregulation of glucocorticoid signalling could play a key role in psychopathology and highlights the promise of pharmacological treatment with glucocorticoids, specifically also given the role of glucocorticoids in emotional memory: experimental studies in healthy participants have demonstrated that elevated cortisol levels caused by glucocorticoid treatment or stress induction before the extinction of fear memory strengthen the consolidation of safety memory (Drexler et al., 2017; Drexler et al., 2018; Merz et al., 2018). These findings appear to translate to clinical populations of PTSD patients (Inslicht et al., 2021), as well as to actual exposure therapy treatment settings (Meuret et al., 2016; Suris et al., 2010; Yehuda et al., 2010). In a first randomized controlled pilot study, which included 24 veterans affected by PTSD, clinical improvement after hydrocortisone augmentation of exposure therapy was associated with greater glucocorticoid sensitivity at baseline (Yehuda et al., 2015a). Importantly, recent work with rodents suggests that glucocorticoids not only enhance the strength of safety memory, but also increase its generalization to other contexts (Roozendaal & Mirone, 2020). A study including healthy males correspondingly indicated that stress induction before extinction learning enhanced the context independence of safety memory (Drexler et al., 2018). These findings show additional potential for glucocorticoids to enhance trauma-focused therapy outcome, as it is common for PTSD patients to experience the return of fear and relapse outside the therapeutic context.

Glucocorticoids such as cortisol provide a mechanism for enhancing extinction processes by acting on brain regions that play key roles in the emotional modulation of memory processes (de Quervain et al., 2017; Roozendaal & McGaugh, 2011). Previous studies have suggested that activation of the dorsal anterior cingulate cortex and anterior insular cortex – brain regions importantly associated with fear expression – at late extinction may indicate incomplete extinction of fear memory, whereas activations in the dorsolateral prefrontal cortex and ventromedial prefrontal cortex – brain regions importantly associated with fear extinction – would indicate a regulatory response that suppresses the expression of fear (Fullana et al., 2018; Picó-Pérez et al., 2019). Of note, effective extinction learning may also be signalled by patterns of functional connectivity between brain networks (Wen et al., 2022). Individuals with PTSD show increased activation in brain regions associated with fear expression and in the hippocampus, corresponding to the notion of weaker and more context-specific extinction learning (Suarez-Jimenez et al., 2019). Higher activations in fear-related networks and lower activations in the dorsolateral prefrontal cortex during emotion regulation tasks were also found to be predictive of poorer PTSD treatment outcome (Fonzo et al., 2017; Rooij et al., 2016). In correspondence, targeting prefrontal areas with transcranial magnetic stimulation can improve extinction learning outcomes (Raij et al., 2018).

Collectively, the aforementioned findings suggest a mechanistic model in which PTSD patients with dysfunctions in glucocorticoid signalling – potentially emerging from epigenetic programming by ELA – insufficiently benefit from PTSD treatments. This group of non-responding and relapsing individuals could potentially benefit from exogenous glucocorticoid administration during extinction learning (De Quervain et al., 2019). The current study integrates an observational study with a pharmacological intervention and functional resonance imaging (fMRI) study to provide a translational effort towards improved and personalized PTSD treatment. In our observational study, we investigate interaction effects of ELA and epigenetic programming of glucocorticoid signalling on HPA-axis regulation and their association with treatment outcome in a group of individuals with PTSD. The pharmacological intervention and fMRI study investigates glucocorticoid-induced enhancement of extinction learning in PTSD patients in a placebo-controlled clinical trial with a crossover design. By leveraging an experimental fear conditioning and extinction task within a virtual environment that uses houses as spatial contexts for the experiences, we will be able to investigate the effect of exogenous glucocorticoids on the context dependency of fear and extinction memories. Overall, we aim to provide proof of concept that HPA-axis dysregulation in individuals with PTSD is related to ELA and associated epigenetic markers of glucocorticoid signalling; that these individuals with PTSD show impairments in extinction learning and altered neural responses; and that this subgroup would particularly benefit from glucocorticoid augmentation of extinction learning through a mechanism of enhancing the strength and generalization of extinction memories.

### 1.1. Research objectives

Objectives of the observational study:

1. Replicate findings of HPA-axis dysregulation in PTSD patients relative to a control group of individuals without PTSD or history of early-life trauma.
2. Investigate associations between history of ELA and HPA-axis dysregulation.
3. Investigate whether epigenetic markers of glucocorticoid signalling (such as methylation status of glucocorticoid-related genes) mediate the association between history of ELA and HPA-axis dysregulation.
4. Exploratively, investigate whether HPA-axis dysregulation and glucocorticoid signalling are associated with treatment response.

Objectives of the pharmacological intervention study:

1. Investigate whether patients with HPA-axis dysregulation show impairments in extinction learning relative to patients without HPA-axis dysregulation, and whether this is accompanied by different neural responses during extinction learning in brain regions associated with fear expression and extinction.
2. Investigate whether the subgroup of PTSD patients with HPA-axis dysregulation particularly benefits from hydrocortisone administration (versus placebo) to improve the strength and generalization of extinction memories.
3. Investigate neural responses during extinction learning in brain regions associated with fear expression and extinction after hydrocortisone administration (versus placebo), that may be associated with improvements in strength and generalization of extinction learning.

## 2. Method

### 2.1. Design

**Figure 1.**
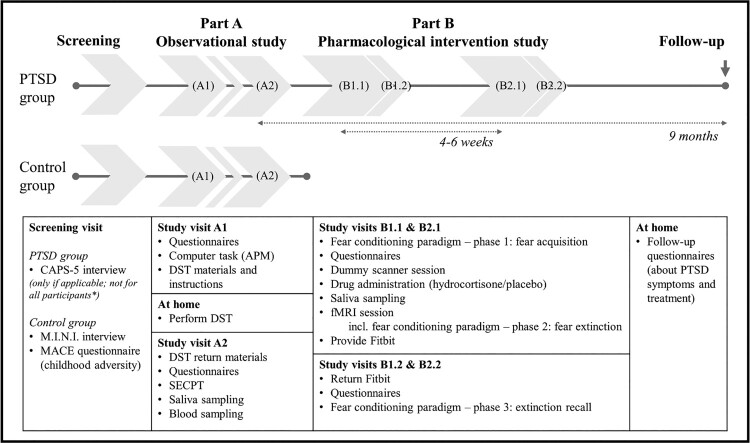
Overview of study procedures. This study consists of two integrated parts. For some participants, the order of study visits is changed to reduce the total duration of participation; these participants complete study visits B1.1 and B1.2 prior to study visits A1 and A2. *Only post-traumatic stress disorder (PTSD) group participants recruited from the general population complete a screening visit; participants recruited from one of the collaborating mental healthcare institutions are included based on a (suspected or preliminary) diagnosis of PTSD for which they received an indication for trauma-focused treatment. APM = Advanced Progressive Matrices; CAPS-5 = Clinician-Administered PTSD Scale for DSM-5; MINI = Mini International Neuropsychiatric Interview; MACE = Maltreatment and Abuse Chronology of Exposure; DST = dexamethasone suppression test; SECPT = socially evaluated cold-pressor test; fMRI = functional magnetic resonance imaging.

This study consists of two integrated parts (Figure 1). The first part, referred to as ‘part A’, is an observational study investigating HPA-axis characteristics. The second part, referred to as ‘part B’, is a randomized placebo-controlled pharmacological intervention and fMRI study with a crossover design, investigating the effect of hydrocortisone administration on extinction learning within a differential fear acquisition, extinction, and extinction recall task. Participants with PTSD can either participate in the entire study – that is, both parts, preferably sequentially, but alternatively in parallel – or only in part A. These PTSD group participants are recruited from collaborating mental healthcare institutions and the general population. A healthy, matched control group without exposure to early-life trauma is recruited from the general population and only participates in part A.

### 2.2. Population

This study aims to include *n *= 160 people with PTSD (part A), half of whom participate in the entire study (*n = *80 for part B). In addition, *n *= 30 control group participants are recruited (part A). All participants must be aged 18–64 years old. Exclusion criteria for both groups are listed in Table 1. More details on criteria assessed during the screening visit and amendments during the trial are listed in the Supplementary Material (S1 and S2). Power calculations are also described in the Supplementary Material (S3).

**Table 1.** Exclusion criteria.

Group	Exclusion criteria
PTSD	General exclusion criteria:
	- Current psychotic or manic episode
	- Benzodiazepine medication: Daily intake of benzodiazepines, or otherwise irregular intake of benzodiazepines (‘when needed’) but unable to withhold intake according to study procedures Exception: low evening doses (sleep medication)
	- For part B only: MRI contraindications
	Criteria assessed during screening session:
	- Does not receive a classification of current PTSD according to clinical interview assessment
Control	General exclusion criteria:
	- Current or past psychiatric disorder
	- Use of psychotropic medication
	- History of childhood maltreatment
	Criteria assessed during screening session:
	- Early-life adversity according to questionnaire assessment
	- (History of) psychiatric disorders according to clinical interview assessment
Both	General exclusion criteria:
	- General learning disability or intellectual disability
	- Body mass index > 35 kg/m^2^
	- Pregnancy or breastfeeding
	- Substance use: Unable or unwilling to withhold recreational drug use and limit alcohol use according to study procedures
	- Nicotine smoking: Unable or unwilling to discuss frequency and timing of smoking according to study procedures
	- Glucocorticoid medication/supplements: Current or recent regular use of systemic corticosteroids (< 1 month ago). For non-systemic corticosteroids, average use of more than once a week and/or no possibility to withhold according to study procedures
	- Hypersensitivity to glucocorticoids
	Criteria only if relevant to study outcomes or feasibility of study procedures:
	- Relevant neurological disorder
	- Severe physical disorder
	- Relevant endocrine disease and/or current or recent endocrine treatment

Note: MRI = magnetic resonance imaging; PTSD = post-traumatic stress disorder; BMI = body mass index.

#### 2.2.1. PTSD group

When recruited from one of the collaborating mental healthcare institutions, the participant should have received a (suspected or preliminary) diagnosis of PTSD for which they received an indication for trauma-focused treatment. Alternatively, participants should have previously received a diagnosis of PTSD and currently still experience considerable PTSD symptoms; current PTSD is then verified by means of a structured clinical interview. The aim is to achieve a prespecified gender distribution, with an acceptable (dis)balance ranging from 40–75% women; that is, 25–60% men and other gender identities. This range is asymmetrical given the disbalance in PTSD prevalence and because of hormonal contraceptive use by women, which may be investigated as a confounder variable.

#### 2.2.2. Control group

The control group is screened for having no history of psychiatric disorders, no history of childhood trauma or maltreatment, and no current use of psychotropic medication (for details see Supplementary Material S1). Control participants will be selected based on age, gender, and education, with the aim of matching the distributions of these demographic variables to the (expected) distributions within the PTSD group.

### 2.3. Pharmacological intervention

In part B of the study, to investigate glucocorticoid effects on extinction learning and brain activity, participants ingest 20 mg hydrocortisone prior to extinction learning in one session, and placebo prior to extinction learning in the other session. The order of medication administration is double-blind and counterbalanced across participants (for details see Supplementary Material S4).

### 2.4. Materials

#### 2.4.1. Clinical and questionnaire assessments

##### 2.4.1.1. Classification of PTSD (PTSD group)

PTSD group participants who are invited by a clinician from a collaborating mental healthcare institution are included based on clinical judgement and according to local organizational methods of PTSD classification (without additional verification within the current study). However, for participants in the PTSD group from the general population, classification of PTSD is assessed with the Dutch version of the Clinician-Administered PTSD Scale for DSM-5 (CAPS-5), which is a structured clinical interview for PTSD (Boeschoten et al., 2018).

##### 2.4.1.2. Screening for psychiatric disorders (control group)

In the control group, current psychiatric disorders are screened for with the Dutch translation of the Mini International Neuropsychiatric Interview (MINI) version 5, a structured clinical interview that is based on the Diagnostic and Statistical Manual of Mental Disorders, 4th Edition (DSM-IV) (Sheehan et al., 1998).

##### 2.4.1.3. PTSD symptoms

PTSD symptoms are assessed with the Dutch version of the PTSD Checklist for DSM-5 (PCL-5), a 20-item self-report questionnaire that assesses symptoms in the past month according to the Diagnostic and Statistical Manual of Mental Disorders, Fifth Edition (DSM-5) (Blevins et al., 2015; Boeschoten et al., 2014).

##### 2.4.1.4. Childhood trauma

Childhood traumatic experiences are assessed with the Dutch translation of Maltreatment and Abuse Chronology of Exposure (MACE), a 52-item self-report questionnaire that assesses exposure to 10 types of childhood trauma and neglect, as well as the ages of exposure (Teicher & Parigger, 2015).

##### 2.4.1.5. Other questionnaires

All clinical and questionnaire assessments are described in the Supplementary Material (S5 and S6).

#### 2.4.2. Biological materials

##### 2.4.2.1. Saliva sampling

Saliva is sampled using Salivette collection devices (Sarstedt, Rommelsdorf, Germany). Each sample is stored at −20°C until biochemical analysis of cortisol levels.

##### 2.4.2.2. Blood sampling

For assessment of DNA methylation levels and genotyping, blood is drawn into one (10 ml) K2EDTA-containing tube. In addition, blood is drawn into two (2.5 ml) PAXgene tubes for determination of mRNA levels. The samples are cooled at +4°C for approximately 45 minutes, then frozen at –20°C for 12–24 hours (overnight) and then stored at –80 to –70°C for long-term storage.

#### 2.4.3. Dexamethasone suppression test

The low-dose dexamethasone suppression test (DST) measures suppressibility of the HPA axis caused by GR-mediated negative feedback by the administration of 0.5 mg dexamethasone (de Kloet et al., 2006). Participants collect saliva samples at waking time and 15, 30, 45, and 60 minutes and 8 hours after waking time on two days at home. In this study, dexamethasone is taken orally at 11 p.m. (23:00 h) on the evening before the second day of the test. Additional instructions ensure the quality of the saliva sample (for details, see Supplementary Material S6). More detailed information on instructional materials and procedures is provided in the Supplementary Material (S7).

#### 2.4.4. Socially evaluated cold-pressor test

The socially evaluated cold-pressor test (SECPT) is a validated stress paradigm that induces robust increases in subjective stress ratings, blood pressure, and salivary cortisol levels (Schwabe et al., 2008; Schwabe & Schächinger, 2018). The SECPT protocol was adapted after consultation with our advice committee of experts – people with personal experience of PTSD – not to include a camera set-up, as this was considered too stressful for certain (social anxiety) subpopulations. For details about its implementation in the current study, see the Supplementary Material (S8).

#### 2.4.5. Fear conditioning and extinction task

**Figure 2.**
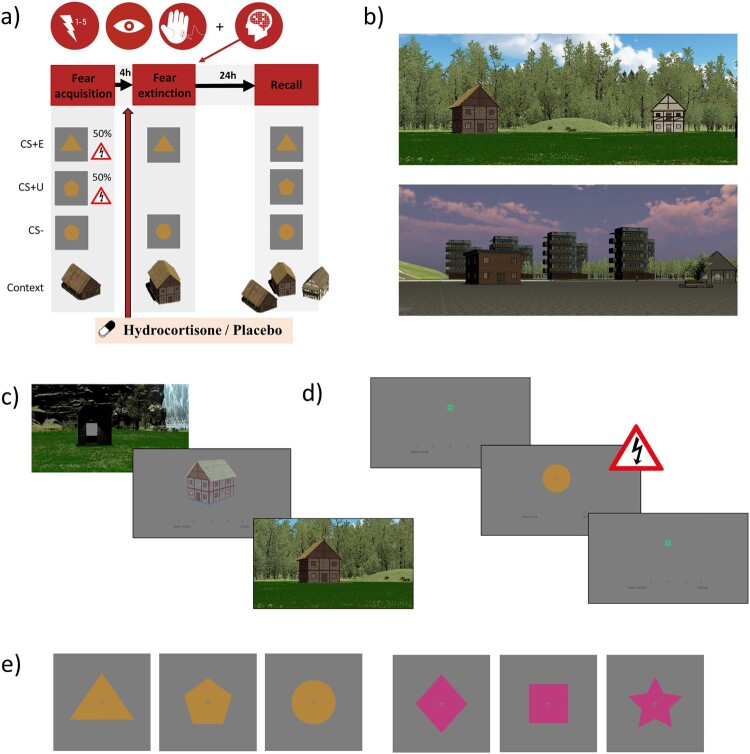
Fear conditioning and extinction task. (a) The task consists of a fear acquisition, fear extinction, and extinction recall task. In this study, hydrocortisone or placebo is administered 45–55 minutes prior to the fear extinction task. This fear extinction task is completed during functional magnetic resonance imaging (fMRI) scanning. Outcome measures are psychophysiological measurements (pupil, skin conductance) and shock expectancy ratings during all tasks, and neuroimaging during the fear extinction task. (b) To accommodate the crossover design of this study, the task has two virtual environments (nature and city environment) with three houses each. These houses function as occasion setters, i.e., ‘contexts’ for the experiences (acquisition, extinction, novel). (c) Task procedures to start a task block. The participant is first instructed to enter a booth. Inside this booth, they are presented with the house to which they should navigate. They should also rate on a five-point scale whether they expect to receive at least one shock within that house in the upcoming block. Entering a booth within the correct house triggers the start of a block. (d) Task procedures within a block, per trial. The participant is presented with a fixation cross before presentation of a conditioned stimulus (CS). During CS presentation, they should rate on a five-point scale whether they expect to receive a shock based on the stimulus that is being presented. At the end of stimulus presentation, depending on the task and trial, negative reinforcement with an electric shock to the fingers may follow. (e) Two sets of three shapes that function as CSs within the paradigm.

To assess the strength and generalization of safety learning within an experimental setting, the current study employs a 2 day differential fear acquisition, extinction, and extinction recall task in a virtual environment (VE) with contexts as occasion setters (Figure 2). This task was adapted from previous studies and subsequently validated in a study with healthy young adults (de Nooij et al., 2021; de Voogd et al., 2020; Wirz et al., 2021). The task consists of two different VEs (natural scene and city scene) to allow repeated measurements within the crossover design of this study. Contexts for experiences are defined by three different houses within each VE. The conditioned stimuli (CSs) are simple geometric shapes and the unconditioned stimulus (US) is an electric shock to the fingers. The task consists of three phases: (1) fear acquisition, (2) fear extinction, and (3) recall. In each phase, the participant navigates within the three-dimensional VE using a joystick. After navigating to one of the houses, the participant is presented with a trial block of a two-dimensional task. During each trial, the participant provides a shock expectancy rating (SER) on a scale from 1 (‘certainly no shock’) to 5 (‘certainly a shock’). Primary outcomes consist of fear-related measures of autonomic nervous system response, particularly the skin conductance response (SCR) and pupil dilation response (PDR); these are assessed during each phase of the task. Further details on the task, experimental set-up, and counterbalancing are outlined in the Supplementary Material (S9).

##### 2.4.5.1. Task phases

1. *Fear acquisition.* Three different CSs are presented in four blocks. Each block consists of four trials per CS, totalling to 12 trials per block. One CS is never followed by shock (CS−), whereas two other CSs (CS+s) are sometimes followed by shock according to a 50% reinforcement schedule. All blocks are completed within the same house; this house is established as the ‘acquisition context’.
2. *Fear extinction.* The CS− and one of the two CS+s are presented in four blocks. Each block consists of four trials per CS, totalling to eight trials per block. Here, the included CS+ is being extinguished (CS+E) as no shocks are administered during this phase. The CS+ that is omitted from this phase remains unextinguished (CS+U). All blocks are completed within the same house, differently from the fear acquisition phase; this house is established as the ‘extinction context’.
3. *Extinction recall.* All three CSs (CS+E, CS+U, and CS−) are presented in six blocks. Each block consists of two trials per CS, totalling to six trials per block. The first two blocks are presented within the ‘extinction context' house, the next two blocks within the ‘novel context' house, and the last two blocks within the ‘acquisition context' house.

##### 2.4.5.2. Practice tasks

In relation to the fear conditioning and extinction task, two practice tasks were developed: one to practise navigation with the joystick and for familiarization with the VE, and one to practise giving scores with the joystick and more generally to practise the required course of actions (for details, see Supplementary Material S9).

#### 2.4.6. Neuroimaging

Details of magnetic resonance imaging (MRI) data acquisition are described in the Supplementary Material (S10).

##### 2.4.6.1. Task-based fMRI: fear extinction

Neural responses associated with extinction learning are assessed during the fear extinction task, as previously described.

##### 2.4.6.2. Task-based fMRI: emotion processing

Neural responses during implicit emotion processing are assessed using an adaptation of an emotional face matching task (Hariri et al., 2002). The current task version was implemented by the DynaMORE consortium (for details, see Wackerhagen et al., 2023). In short, grey-scale faces with angry and fearful expressions that should be matched are presented during the emotion condition, whereas in the shape condition, geometric shapes (circles, horizontal ellipses, and vertical ellipses) that should be matched are presented. The task consists of eight blocks of six trials that alternate between conditions.

##### 2.4.6.3. Resting-state fMRI

For resting-state scans, participants are presented with a fixation cross (white cross on black background). They are instructed to keep their eyes open and have their thoughts flow freely, but refrain from repetitive mental activities such as counting.

### 2.5. Procedure

Additional information on study procedures can be found in the Supplementary Material (S11).

#### 2.5.1. Recruitment

Participants from the PTSD group are recruited via Pro Persona and Kairos, which are collaborating mental healthcare institutions located in and around Nijmegen. In addition, following approval of a study amendment, participants from the PTSD group are also recruited from the general population via (social media) advertisements, distribution of posters and flyers, and personal and professional networks. PTSD group participants are directed to a study website with short informational videoclips and other information. They are personally contacted and complete a telephone screening to assess inclusion and exclusion criteria. Participants from the control group are recruited from the general population via (social media) advertisements and distribution of posters and flyers, and receive information and register via a study website for the control group.

#### 2.5.2. Participant inclusion

All participants provide informed consent at their first visit. This is followed by verification and registration of inclusion and exclusion criteria, and collection of general information.

#### 2.5.3. Screening visit

##### 2.5.3.1. Screening visit for PTSD group

The screening visit only applies to PTSD patients who were recruited from the general population. Based on the preference of the participant and feasibility, this visit can be scheduled on location or as an online visit. During this visit, an online questionnaire and CAPS-5 clinical interview are administered to the participant. The participant is immediately informed about inclusion or exclusion in this study, depending on whether they meet classification criteria for PTSD or not.

##### 2.5.3.2. Screening visit for control group

The participant completes the MACE as an online questionnaire, followed by a MINI assessment. Based on their results, the participant is immediately informed about inclusion in or exclusion from this study (for details, see Supplementary Material S1).

#### 2.5.4. Observational study (Part A)

##### 2.5.4.1. First visit (A1)

During this visit, the participant completes MACE (if not previously completed) and several other online questionnaires/assessments. Subsequently, they receive materials and in-person instructions for the DST procedure (for details, see Supplementary Material S7).

##### 2.5.4.2. Second visit (A2)

This visit is scheduled in in the afternoon [SECPT from 1 p.m. (13:00 h)] to avoid any influence of higher morning cortisol levels. The researcher receives the materials for the DST procedure from the participant and evaluates the procedure. Then, the participant completes the PCL-5 and several other online questionnaires before participating in the SECPT procedure. Here, saliva samples are collected at 15, 30, 45, 60, 75, and 90 minutes after the onset of the stress procedure. Lastly, venous blood is sampled by a trained research nurse at the Radboud University Medical Center.

#### 2.5.5. Pharmacological intervention and fMRI study (Part B)

**Figure 3.**
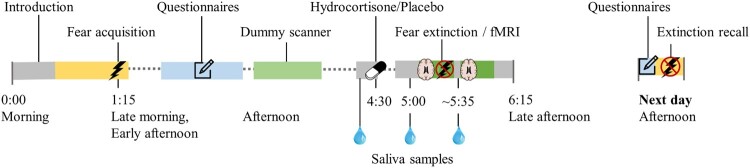
Timeline of study procedures per session in the pharmacological intervention and functional magnetic resonance imaging (fMRI) study. The first study visit typically starts around 10 or 11 a.m. (10:00 or 11:00 h), and the next-day visit typically starts around 3.15 or 4.15 p.m. (15:15 or 16:15 h).

Owing to the crossover design, this study part consists of two sessions that are scheduled with a 4–6 week interval. The sessions are identical, except for the type of drug administered (hydrocortisone or placebo) and the version of the fear conditioning and extinction task (different VE with different set of CSs). Each session includes two visits to the Donders Centre for Cognitive Neuroimaging on subsequent days (Figure 3). Before each visit, participants receive instructions for quality assurance of data (for details, see Supplementary Material S6).

##### 2.5.5.1. MRI visit (B1.1 and B2.1)

In the psychophysiology laboratory, the participant completes the two practice tasks (see Section 2.4.5) in the presence of the researcher. If more practice is needed, a practice test is repeated. The intensity of electric shocks is determined by a calibration procedure (for details, see Supplementary Material S9). The participant then performs the fear acquisition task with psychophysiological assessment (pupil, skin conductance, heart rate). After a lunch break, the participant completes several online questionnaires, followed by a practice session in the dummy MRI laboratory and a second break. Subsequently, they provide a baseline saliva sample, which is followed by the oral intake of the study medication (indistinguishable capsule with 20 mg hydrocortisone or placebo). During the experimental set-up for the MRI session, the participant provides another saliva sample 30 minutes after medication administration. Heart rate and respiratory data are collected throughout the MRI session. The first part of the MRI session consists of a field map and task-based fMRI during fear extinction (45–55 minutes after medication administration) with psychophysiological assessment (pupil, skin conductance, heart rate). This is followed by a structural scan (T1-weighted during the first session; diffusion tensor imaging during the second session). During a short break, another saliva sample is provided (60–75 minutes after medication administration). The second part of the MRI session consists of resting-state fMRI with field mapping, and task-based fMRI during emotion processing with field mapping.

##### 2.5.5.2. Next-day visit (B1.2 and B2.2)

The participant completes online questionnaires and returns to the psychophysiology laboratory to complete the extinction recall task with psychophysiological assessment (pupil, skin conductance, heart rate). This visit is always scheduled the next day in the late afternoon, with an interval of at least 24 hours between the start of the fear extinction task and extinction recall task.

#### 2.5.6. Follow-up survey

An online survey is sent out only to participants from the PTSD group 9 months after the last visit of the observational study (i.e. study visit A2) to assess, if applicable, their response to treatments (i.e. treatment as usual which is unrelated to the current study). This online survey includes the PCL-5 to assess PTSD symptomatology, and questions about receiving psychotherapy and/or pharmacological treatment for PTSD and other psychiatric disorders. The control group was screened to be from a healthy population and as such is not expected to participate in any form of psychiatric treatment; hence, they do not participate in the follow-up survey.

#### 2.5.7. Additional patient data

Participants recruited from a collaborating mental healthcare institution provide consent that the study team may request specific types of information about them from their mental healthcare institution. This includes results from CAPS-5 assessments, which, according to harmonized clinical practice protocols, should be administered prior to trauma-related treatment.

#### 2.5.8. Reimbursement

Participants are offered a financial reimbursement for their time investment and effort, of €92 for the observational study, €130 for the pharmacological intervention study, and €10 for completion of the follow-up survey. When a participant is excluded following a screening visit or discontinues their participation, they are offered a reimbursement equivalent to their contributions. In addition, participants receive compensation for travelling expenses.

### 2.6. Statistical analysis

Mitigation plans to adapt statistical analyses if extension of the data collection period is no longer feasible and the planned sample size is not achieved are described in the Supplementary Material (S12).

#### 2.6.1. Observational study

##### 2.6.1.1. Primary outcome measures

HPA-axis dysregulation is investigated with two procedures by repeated sampling of saliva. These saliva samples will be analysed for cortisol levels using a competitive electrochemiluminescence immunoassay. For the DST, the main parameter of interest is suppression of the cortisol awakening response (CAR) based on the area under the curve (AUC) of normal CAR and dexamethasone-suppressed CAR, calculated as (1 − AUC_DEX_/AUC_CAR_) × 100%. For the SECPT, the main parameter of interest is the AUC with respect to increase (AUC_i_) in the cortisol stress response (Pruessner et al., 2003). Other variables and parameters from both procedures will also be investigated. ELA is assessed with the MACE questionnaire, which provides scores on 10 subscales of abuse and neglect that add up to a total scaled score and a multiple trauma score. It also provides information on the ages of exposure. Blood samples will be analysed for DNA methylation status, genotyping, and mRNA levels. Glucocorticoid signalling is primarily indicated by methylation of glucocorticoid-related genes, including *NR3C1* and *FKBP5*, but mRNA levels and genotype interactions will also be assessed. Treatment response is operationalized as the reduction of self-reported PTSD symptoms according to PCL-5 questionnaire assessments.

##### 2.6.1.2. Statistical modelling

1. Replicate findings of HPA-axis dysregulation in PTSD patients relative to a control group of individuals without PTSD or history of early-life trauma.

Statistical analyses of HPA-axis dysregulation in the PTSD group versus the control group will be tested using analyses of variance (ANOVAs), with group as the predictor of interest, and inclusion of age and gender as covariates. False discovery rate (FDR) correction will be applied over the number ANOVA tests with different HPA-axis dysregulation outcome variables. Furthermore, model extensions will explore additional covariates such as lifestyle factors (smoking, alcohol use, and drug use), concurrent psychiatric symptoms, and (female) menstrual cycle.

2. Investigate associations between history of ELA and HPA-axis dysregulation.

Associations between ELA and HPA-axis dysregulation will be investigated in the PTSD group with linear regression models, with the inclusion of age and gender as covariates. FDR correction will be applied over the number of regression models with different HPA-axis dysregulation outcome variables and/or different ELA predictor variables. Additional linear regression models will explore additional covariates such as other relevant task variables (e.g. SECPT stress ratings, duration of hand in water), lifestyle factors (smoking, alcohol use, and drug use), concurrent psychiatric symptoms, and (female) menstrual cycle.

3. Investigate whether epigenetic markers of glucocorticoid signalling (such as methylation status of glucocorticoid-related genes) mediate the association between history of ELA and HPA-axis dysregulation.

As a validation check of the proposed epigenetic mechanism, it will first be ascertained that for both genes *NR3C1* and *FKBP5* (and potentially other genes of interest), DNA methylation inversely correlates with mRNA levels in the blood. This will be tested with Pearson or Spearman correlation tests. Linear regression models will then investigate the associations between ELA and DNA methylation at *NR3C1* and *FKBP5* in the PTSD group, with the inclusion of age and gender as covariates. For *FKBP5*, we will take into account polymorphisms of SNP rs1360780 (minor risk allele T versus homozygous C-allele carriers) by specification of an ELA-by-genotype interaction effect, because of previously reported allele-specific gene–childhood effects (Klengel et al., 2013). FDR correction will be applied over the number of regression models with different DNA methylation outcome variables and/or different ELA predictor variables. The association between DNA methylation status and HPA-axis dysregulation will also be investigated with linear regression models, with the inclusion of age and gender as covariates. FDR correction will be applied over the number of regression models with different HPA-axis dysregulation outcome variables and/or different DNA methylation predictor variables. Additional linear regression models will explore additional covariates, such as other relevant task variables (e.g. SECPT stress ratings, duration), lifestyle factors (smoking, alcohol use, and drug use), concurrent psychiatric symptoms, and (female) menstrual cycle. If appropriate, regression models will be followed up by mediation models to directly test mediation of the association between ELA and HPA-axis dysregulation by methylation status of glucocorticoid-related genes.

4. Exploratively, investigate whether HPA-axis dysregulation and glucocorticoid signalling are associated with treatment response.

In the PTSD group, functional markers of HPA-axis dysregulation associated with treatment response will be examined with mixed-effects linear regression models. Since the PTSD group will have experienced heterogeneous courses of treatment, a specific analysis plan will be drafted after exploration of the frequency and variety of treatments within the sample.

#### 2.6.2. Pharmacological intervention and fMRI study

##### 2.6.2.1. Primary outcome measures

Successful fear acquisition, as indicated by a higher SER for the CS+s in comparison to CS− in the last block of the fear acquisition task, is a prerequisite for the assessment of extinction learning. During the fear extinction task, fear extinction should be indicated by decreasing SER, showing no expectancy of shock in the last block of this task. Fear acquisition and fear extinction will also be signalled by psychophysiological outcome measures (SCR and PDR). Neuroimaging during the fear extinction task investigates pharmacological effects on neural responses associated with fear extinction learning. Ultimately, the strength and generalization of the fear and extinction memories are assessed in the extinction recall task during recall of these memories upon presentation of the CSs in different contexts. Here, the primary outcome variables are SCR, PDR, and SER. Details of psychophysiological analyses are described in Supplementary Material S13. More specifically, increased strength of extinction memory should be indicated by smaller differential responses within the extinction context, whereas increased generalization of extinction memory should be indicated by a context interaction effect showing transfer of extinction to novel and/or acquisition contexts. In addition, effects of HPA-axis dysregulation are investigated to investigate their role in glucocorticoid effects on extinction recall. Relative suppression of the CAR from the DST is prespecified as the primary outcome measure, and based on a median split, the PTSD group will be divided over two subgroups. Grouping based on other HPA-axis outcome variables will be regarded as secondary and subjected to appropriate multiple-comparison corrections. Planned MRI (preprocessing) analyses are discussed in the Supplementary Material (S14).

##### 2.6.2.2. Statistical modelling

1. Investigate whether patients with HPA-axis dysregulation show impairments in extinction learning relative to patients without HPA-axis dysregulation, and whether this is accompanied by different neural responses during extinction learning in brain regions associated with fear expression and extinction.

This objective will be investigated with data from placebo sessions only. At the psychophysiological level, we expect HPA-axis dysregulation to be associated with lower strength of the extinction memory, indicated by lower differential responses for CS+U vs CS+E and/or higher differential responses for CS+E vs CS− during the extinction recall task. This will be modelled with a two-way ANOVA of averaged differential responses during the first block, which is within the extinction context and contains two presentations of each CS. Higher generalization of the extinction memory for the HPA-axis normalized group would be indicated by a context interaction effect that shows less renewal of fear, i.e. a lower increase of differential effects upon stimulus presentations within the novel and acquisition contexts. These comparisons may be affected by an overall attenuation of fear responses due to re-extinction over the course of the extinction recall task. This effect will be investigated with a 2 (group) by 3 (context) mixed ANOVA of averaged differential responses during the first block of each context. Both models will include the covariate order of medication administration, and, if appropriate, the covariates age and gender. Outcome measures SCR, PDR, and SER will be included in separate models, but since these measures of conditioning are complementary, no multiple comparisons correction will be applied. At the level of neuroimaging, differences in extinction learning signalled during the fear extinction task could be underlying the differences at the behavioural level. Here, we would expect different neural responses in brain regions and networks associated with (context-dependent) extinction learning for patients with HPA-axis dysregulation, which could also be associated with less successful recall of the extinction memory.

2. Investigate whether the subgroup of PTSD patients with HPA-axis dysregulation particularly benefits from hydrocortisone administration (versus placebo) to improve the strength and generalization of extinction memories.

To answer the main objective of the pharmacological intervention study, the aforementioned psychophysiological models will be extended with data from the hydrocortisone sessions (thus adding a within-subjects category for medication). Period effects may follow from the crossover design and will be accounted for by defining a medication-by-order interaction effect. Based on previous research, we hypothesize a group-by-medication interaction effect, indicating greater strength and/or generalization of the extinction memory following administration of hydrocortisone, that is most pronounced for the HPA-axis dysregulation group.

3. Investigate underlying neural mechanisms of glucocorticoid administration (versus placebo) to improve extinction learning.

Recruitment of neural networks associated with (context-dependent) extinction learning for patients with HPA-axis dysregulation (see point 1 above) would be expected to enhance following hydrocortisone administration. Pharmacological effects and their potential interactions with HPA-axis dysregulation are primarily investigated during the extinction learning task, but will also be supported by the following analyses: (1) amygdala (and salience network) reactivity to the emotion processing task, which we expect to be reduced after hydrocortisone administration; and (2) resting-state connectivity in networks associated with (context-dependent) extinction learning – ventromedial prefrontal cortex–amygdala connectivity, in particular – which we expect to be enhanced after hydrocortisone administration.

## 3. Discussion

### 3.1. Study progress

The start of this study was delayed as a consequence of the coronavirus disease 2019 (COVID-19) pandemic, after which its first participant was included in December 2021. Subsequently, the current study faced further delays in its progress owing to challenges in recruitment of the PTSD group that were exacerbated by long-term effects of the COVID-19 pandemic and institutional reorganizations at collaborating mental healthcare institutions. As of March 2024, the observational study has completed study visits for *n *= 55 participants from the PTSD group and *n *= 23 participants from the control group. Of the PTSD group, *n = *19 participants have additionally completed the pharmacological intervention study. This study has undergone several amendments to manage these challenges. Most importantly, initially all participants were included via collaborating mental healthcare institutions; in order to increase inclusion rates, inclusion of PTSD group participants from the general population started in January 2023, followed by large advertising campaigns from May 2023.

### 3.2. Collaborations

The current project is being conducted by a multidisciplinary, multicentre team. First, close collaboration with a patient federation as well as a committee of people with personal experience of PTSD has provided invaluable insights and recommendations for conducting this research. Secondly, this project runs parallel to a fundamental research project with rodents, which is carried out by the same team and tackles these questions from a different approach. The research project includes two rat lines with differential HPA-axis regulation and employs a similar fear acquisition, fear extinction, and extinction recall task. Here, findings from the rodent studies may also inform novel hypotheses or lead to substantiated adaptations or additions to planned analyses. The patient and rodent research projects will have complementary strengths and weaknesses. Animal research is vital to understand the causal role of the neural mechanisms mediating the effects of enhanced HPA-axis feedback regulation and exogenous glucocorticoids on the strength and generalization of extinction memories.

### 3.3. Strengths and limitations

The current study protocol describes an integrated and comprehensive study, which is conducted by a multidisciplinary team. The application of experimental tasks and procedures as part of assessments to a clinical group of PTSD patients allows mechanistic insights with a high potential for translation to clinical practice. As such, this study may pave the way for future clinical studies that investigate personalized PTSD treatment. The current study also has some limitations. For example, self-report questionnaires are used to assess symptomatology at follow-up. These are considered less reliable than structured clinical interviews such as CAPS-5, but assess a greater range of symptoms while placing less burden on participants (and may thus also show increased response rates). The current study only includes a CAPS-5 screening visit for PTSD participants recruited from the general population. PTSD participants who are recruited from local health mental institutes do not participate in this screening visit; here, we rely on harmonized clinical practice protocols for PTSD classification and treatment indication that include the administration of CAPS-5. Although PTSD classification is not verified by the study team prior to participation (owing to logistical challenges), participants provide consent so that we may receive this information from the local health mental institutes at a later stage. Of note, necessary amendments during the collection of study data resulted in additional limitations. Inclusion of PTSD group participants from the general population may lead to a selection bias of participants with chronic/complex PTSD who have particular interest in our stress system dysregulation hypotheses. It also increases the subgroup of participants that receives no PTSD treatment shortly after study participation and otherwise increases variability in treatment trajectories. This complicates the utility of treatment outcome follow-up measurements, but was necessary to increase inclusion rates.

## 4. Conclusion

Proof of concept that exogenous hydrocortisone can improve safety learning for a subgroup of PTSD patients – which could be provided by this study – will pave the way for a personalized treatment approach for PTSD. In the future, HPA-axis dysregulation could be used as a biomarker for deficits in safety learning processes and may then indicate that treatment may benefit from a pharmacological treatment enhancer. In line with previously established effects of glucocorticoids on emotional learning, the outcomes of this study may corroborate the utility of using hydrocortisone as a safe pharmacological treatment enhancer in trauma-focused treatments such as exposure therapy (de Quervain et al., 2017; De Quervain et al., 2019).

## Supplementary Material

20240419 CovoS protocol paper supplements v5.pdf

## Data Availability

The data sets generated during the current study will be made available in the Donders repository (www.data.donders.ru.nl).
