# Supplementary material for: Exogenous glucocorticoids to improve extinction learning for post-traumatic stress disorder patients with hypothalamic–pituitary–adrenal-axis dysregulation: a study protocol description
Source: Eur J Psychotraumatol. 2024 Jul 8;15(1):2364441. doi: 10.1080/20008066.2024.2364441 (PMC11232644; doi:10.1080/20008066.2024.2364441)
Supplement: 20240419 CovoS protocol paper supplements v5.pdf [file ZEPT_A_2364441_SM3585.pdf]

Supplementary materials

Exogenous glucocorticoids to improve extinction learning for PTSD patients with HPA-axis dysregulation: a study protocol description

Index

S1. Screening criteria for the control group 3

Psychiatric disorders 3

Childhood trauma or maltreatment 3

S2. List of amendments during the trial 5

S3. Power calculations 6

Observational study 6

Pharmacological intervention and fMRI study 6

S4. Counterbalancing 7

S5. Clinical and questionnaire assessments 8

Classification of PTSD (PTSD group) 8

Screening for psychiatric disorders (control group) 8

PTSD symptoms 8

Childhood trauma 8

Depressive symptoms 8

Anxiety symptoms 8

Dissociative symptoms 8

Substance use 8

Sleep 8

Affective state 9

Lifetime trauma 9

Socio-economic status 9

Logical reasoning 9

Menstrual cycle 9

Compliance 9

General information 9

S6. Agreements and instructions for quality assurance 10

S7. Dexamethasone suppression test 11

Rationale 11

Materials 11

Overview of instructions 11

Personal plan 11
Logbook 12
Background information 13
Pill reminder app 13
Procedure 13
Study visit A1 13
Study visit A2 15
S8. Socially evaluated cold-pressor test 16
S9. Fear conditioning and extinction task: additional information and experimental setup 17
Task implementation 17
Task configuration 17
Experimental setup 17
Shock calibration procedure 17
Participant instructions 17
Calibration procedure 18
Habituation 19
Practice tasks 20
Familiarization task 20
Scoring practice task 20
S10. MRI data acquisition 21
S11. Additional information on study procedures 22
First visit (A1) 22
Second visit (A2) 22
MRI visit (B1.1 and B2.1) 22
Next-day visit (B1.2 and B2.2) 22
Additional patient data 22
S12. Alternative analyses 23
Observational study 23
Pharmacological intervention study 23
S13. Psychophysiological analysis 24
S14. MRI analysis 25
References 26

S1. Screening criteria for the control group

Psychiatric disorders

In the healthy control group without early life trauma exposure, participants are excluded in case of self-reported (history of) psychiatric disorders or current use of a psychotropic medication. Additionally, psychiatric disorders are screened for with the Dutch translation of Mini International Neuropsychiatric Interview (M.I.N.I) version 5, a structured clinical interview that is based on DSM-IV [1]. Table S1 details the psychiatric disorders that are screened with the M.I.N.I.

Table S1. Psychiatric disorders that control group participants are screened for using the M.I.N.I, including whether this is being evaluated for lifetime, or only for the current situation.

**Section**	**Psychiatric Disorder**	**Period**
A	Depressive episode	Lifetime
B	Dysthymia	Current
C	Suicidality	Lifetime
D	(Hypo-)manic episode	Lifetime
E	Panic disorder	Lifetime
F	Agoraphobia	Current
G	Social phobia	Current
H	Obsessive-compulsive disorder	Current
I	Posttraumatic stress disorder	Current
J	Alcohol dependency	Current
K	Substance dependency and abuse	Current
L	Psychotic disorders and mood disorders with psychotic features	Lifetime
M	Anorexia Nervosa	Current
N	Bulimia Nervosa	Current
O	Generalized anxiety disorder	Current

Childhood trauma or maltreatment

Childhood traumatic experiences are assessed with the Dutch translation of Maltreatment and Abuse Chronology of Exposure (MACE), a 52-item self-report questionnaire that assess exposure to ten types of childhood trauma and neglect as well as ages of exposure [2]. This questionnaire was administered to both groups in the current study, however for the control group it was used to screen for non-trauma exposed individuals. For this purpose, previously determined cutoff scores per subscale for clinically-significant exposure levels were considered to be too lenient. Therefore, subscale cutoff scores were altered to allow one item less, resulting in cutoff scores per subscale as reported in Table S2. For control group participants, exclusion follows when scoring on or above the cutoff score on one or more subscales.

Table S2: Cutoff scores per MACE subscale (reported as number of applicable items) that are applied in the current study for screening of control group participants.

**Subscales**	**Cutoff score (# items)**
Familial and non-familial sexual abuse	1
Parental verbal abuse	2
Parental non-verbal abuse	3
Parental physical maltreatment	3
Witnessing physical abuse between parents	1
Witnessing Abuse toward sibling	1
Peer verbal abuse and ostracism	3
Peer physical bullying	1
Emotional neglect	1
Physical neglect	1

S2. List of amendments during the trial

For the current study, several substantial amendments were filed when data collection was already ongoing. These have changed the study procedures, usually allowing more flexibility in recruitment or implementation of the study procedures. Table S3 lists these amendments chronologically, including date of approval and rationale.

Table S3. Substantial study amendments at the time of ongoing data collection.

Date of approval	Original	After amendment	Rationale
11-01-2022	- Inclusion criterion: aged 18-55 years<br>- Exclusion criteria for the entire study	- Inclusion criterion: aged 18-64 years<br>- Some exclusion criteria only for part A	Increase size of study population to improve inclusion rates
01-08-2022	- Exclusion criterion: Body Mass Index > 30	- Exclusion criterion: Body Mass Index > 35	Increase size of study population to improve inclusion rates
05-10-2022	- Study participation starts with part A<br>- Exclusion at start of trauma-focused treatment	- Study participation can start with study visit B1.1 and B1.2 prior to the study visits of part A<br>- Exclusion after three trauma-focused treatment sessions	Limit logistical constraints regarding planning of study visits; increase possibility of study participation; prevent forced drop-out of participants when they start treatment
25-01-2023	- Integrated study with one participant information brochure<br>- Only route of inclusion via collaborating mental health institutions<br>- Follow-up survey is sent 3 months after start of trauma-focused treatment<br>- Exclusion criterion: endocrine disease and/or current or recent endocrine treatment (<1 month ago)	- Part A and part B are being presented more separately in recruitment materials, and have separate participant information brochures<br>- Additional route of inclusion via general population, following screening with a clinical interview<br>- Follow-up survey is sent 9 months after study visit A2.<br>- Exclusion criterion: relevant endocrine disease and/or current or recent endocrine treatment (<1 month ago), only if this is expected to affect participant safety or study outcome variables	More suitable participant information materials for participants who only participate in part A of the study; increase size of study population to improve inclusion rates; limit logistical constraints regarding sending of follow-up surveys; general interval for follow-up survey regardless of route of inclusion and treatment plan
17-04-2023	- Exclusion criterion: recent use of corticosteroids (<1 month ago)	- Exclusion criterion: recent use of systemic corticosteroids (<1 month ago), and in case of non-systemic corticosteroids, average use of more than once a week and/or no possibility to refrain usage the day before and the day of the visit	Increase size of study population to improve inclusion rates

S3. Power calculations

All power calculations were conducted using G*Power 3.1.9.4 [3]. They were performed for α=0.05 and with the planned study sample size, unless otherwise reported. Planned sample sizes were based on expected effect sizes (based on previous studies) and relevant effect sizes for primary outcome variables.

Observational study

The following power calculations were performed for the observational study:

1. First objective: a power calculation was based on a meta-analysis that reported an association between ELA and HPA-axis dysregulation analyzing samples from a healthy population [4]. According to this calculation (correlation point biserial model, one-tailed, r = -0.195), this study will achieve a power = 0.80 for the association between ELA and HPA-axis dysregulation. It is expected that the effect may be greater within a clinical sample of PTSD patients.
2. Second objective:
    a. A first power calculation was based on a study that reported an association between NR3C1 methylation levels and HPA-axis dysregulation [5]. According to this calculation (correlation point biserial model, one-tailed, r=-0.198), this study will achieve power = 0.82 for the association between NR3C1 methylation and HPA-axis dysregulation.
    b. A second power calculation was based on a study that reported allele-specific ELA effects on FKBP5 methylation levels [6]. According to this calculation (correlation point biserial model, one-tailed, r=-0.273), with the inclusion of an (expected) sample of at least n≥100 risk allele carriers (59-74% of the participants), the correlation between ELA and FKBP5 methylation could be reliably detected with power ≥ 0.88.
3. Third objective: a power calculation was based on a study that reported HPA-axis dysregulation in a PTSD group versus a control group [7]. According to this calculation (independent sample t-test, one-tailed, d=0.668, n1=160), this study will achieve power = 0.96 with a sample size of n=30 control participants.

Pharmacological intervention and fMRI study

The power calculation for the pharmacological intervention and fMRI study was performed based on a previous clinical study that investigated the effect of hydrocortisone versus placebo administration before virtual environment exposure therapy sessions within a different group of stress-related disorders patients [8]. A power calculation (repeated measures ANOVA, *r*[repeated measures]=0.3, non-sphericity correction ε=1, power=0.8) based on the effect size of their physiological outcome measure (differential skin conductance response) indicated that a sample size of *n*=33 would be required to detect a medication effect across the sample. For extension of the statistical model with an HPA-axis grouping variable, a sensitivity analysis (repeated measures ANOVA within-between interaction effect, *r*[repeated measures]=0.3, non-sphericity correction ε=1, power=0.8) indicated that a sample size of *n*=80 would be sufficient to detect a small-to-medium effect size of f=0.188. \

S4. Counterbalancing

A single counterbalancing scheme incorporates counterbalancing of the order of medication administration, to which the executive researchers are blinded, with (deblinded) task configuration settings. The task configuration settings included in the counterbalancing scheme are (2) order of virtual environments (VE) × (6) order of conditioned stimulus presentation during the recall phase of the paradigm. The counterbalancing scheme was achieved with an script in R that was thoroughly checked with test runs, and finally executed with a random seed that was only known to deblinded principal investigators. The script would then produce a complete (deblinded) counterbalancing scheme, but also a blinded version that only contained task configuration features. This information was used to create one configuration file per randomization number for the VR fear conditioning and extinction task.

More specifically, the package “RandomizeR” [9] was used to create fourteen sequences of six items that would determine the first medication administration, with always three times each medication (for example: hydrocortisone, hydrocortisone, placebo, hydrocortisone, placebo, placebo). Independently from this, task configuration features were randomly shuffled within blocks, resulting six blocks of twelve rows (with all 2 × 6 combinations) and two blocks of six rows. Each block of six rows, and within each block of twelve rows, each upper and lower block of six rows, would always contain every order of stimulus presentation once, and every order of VE three times. Subsequently, the randomized blocks and sequences, still separated task configuration features and medication administration, were then combined into one counterbalancing scheme. First, task configuration blocks were allocated to one of the medication types (hydrocortisone or placebo). Then, starting from the medication sequences, each occurrence was combined with the subsequent row of an allocated task configuration block. For combinations with task configuration blocks of twelve rows, the resulting block of twenty-four rows is saturated with (2) medications x (2) VE x (6) stimulus presentations possibilities. In addition, the method assured that each block of six rows always contains equal rows with regard to medication, and each block of twelve rows contains equal rows with regard to VE and stimulus presentation.

During data collection, allocation of randomization numbers is stratified to the gender of participants, with one saturated block of twenty-four rows being assigned to females, one saturated block of twenty-four rows being assigned to males; each other block of twelve or six rows will preferably be assigned specifically to females, males, or other gender identities.

S5. Clinical and questionnaire assessments

<u>Classification of PTSD (PTSD group)</u>

PTSD group participants that are invited by a clinician from a collaborating mental healthcare institution are included based on clinical judgement and according to local organizational methods of PTSD classification (without additional verification within the current study). However, for participants in the PTSD group from the general population, classification of PTSD is assessed with the Dutch version of Clinician-Administered PTSD Scale for DSM-5 (CAPS-5), which is a structured clinical interview for PTSD [10].

<u>Screening for psychiatric disorders (control group)</u>

In the control group, past and current psychiatric disorders are screened with the Dutch translation of Mini International Neuropsychiatric Interview (M.I.N.I) version 5, a structured clinical interview that is based on DSM-IV [1].

<u>PTSD symptoms</u>

PTSD symptoms are assessed with the Dutch version of the PTSD Checklist for DSM-5 (PCL-5), a 20-item self-report questionnaire that assesses symptoms in the past month according to DSM-5 [11, 12].

<u>Childhood trauma</u>

Childhood traumatic experiences are assessed with the Dutch translation of Maltreatment and Abuse Chronology of Exposure (MACE), a 52-item self-report questionnaire that assess exposure to ten types of childhood trauma and neglect as well as ages of exposure [2].

<u>Depressive symptoms</u>

Depressive symptoms are assessed with the Dutch translation of the Inventory of Depressive Symptomatology – Self Rated (IDS-SR), a 30-item self-report questionnaire that assesses severity of depressive symptomatology in the past week [13].

<u>Anxiety symptoms</u>

Anxiety symptoms are assessed with the Dutch translation of the Beck Anxiety Inventory (BAI), a 21-item self-report questionnaire that assesses severity of anxiety symptoms in the past week [14].

<u>Dissociative symptoms</u>

Dissociative symptoms are assessed with the Dutch translation of the Dissociative Experiences Scale taxon (DES-T) [15], which consists of 8 self-report items about dissociative experiences that are rated on frequency with an 11-point percentage scale (0% = never, 100% = always).

<u>Substance use</u>

Use and abuse of alcohol, drugs and nicotine are assessed in an online questionnaire based on the first section the original Dutch version of the interview Measurements in the Addictions for Triage and Evaluation (MATE-nl) version 2.1, which lists use of different substances in the past month and total years used [16].

<u>Sleep</u>

Sleep is subjectively assessed with the Dutch version of the Pittsburgh Sleep Quality Index (PSQI) which measures sleep quality and disturbances in the past month [17, 18], and a self-adapted version

that applies to sleep quality of the previous night only. Additionally, Fitbit Inspire 2 devices are used to objectively and non-invasively measure sleep [19].

Affective state

Affective state is assessed with the Dutch version of the Positive and Negative Affect Schedule (PANAS), which consists of two 10-item mood scales regarding positive and negative affect [20]. A short (unvalidated) assessment of current affective state comprises of four questions with 7-point scales (1 = strongly disagree, 7 = strongly agree) about feeling fit, feeling well-rested, being enthusiastic, and being nervous. Subjective feelings of stress are assessed with stress ratings on a 10-point scale following the question: "how stressed do you feel at this moment?" (1 = "not stressed at all", 10 = "extremely stressed").

Lifetime trauma

Lifetime traumatic experiences are assessed with the Dutch version of Life Events Checklist for *DSM-5* (LEC-5) [21]. This is a self-report questionnaire that assesses exposure to stressful life events, including age of exposure, whether this was experienced and/or witnessed, and once or more often, and a 10-point scale rating of reliving this experience in the past month.

Socio-economic status

Subjective socio-economic status (SES) is assessed with a ten strung "social ladder", once for themselves, and once for the family they grew up in [22]. Questions regarding parent education, own education, and current income approximate SES objectively. Poverty until sixteen years old and in the current situation is assessed with questions regarding sufficient money for necessary housing, clothes, food and medical care.

Logical reasoning

Logical reasoning is assessed by a computerized 8-item short form of the Raven Advanced Progressive Matrices task [23]. This task is included to complement information about education in characterizing our samples with regard to approximate cognitive ability.

Menstrual cycle

This study included questions about the use of contraceptives, current phase of the menstrual cycle and menopause.

Compliance

This study included questions about compliance to participant agreements and instructions for quality assurance of data (see Supplementary Materials S6).

General information

Upon inclusion in this study, participants were asked about their age, gender, year of birth, education, employment situation/work-related activities, previous PTSD treatments (PTSD group), use of medications, handedness and vision.

S6. Agreements and instructions for quality assurance

Participants receive instructions in order to assure quality of measurements, following agreements that were listed in the participant information brochure. These agreements apply to study visits A2 and all study visits of part B, and are listed below:

- Intake of caffeine:
  - Refrain on the days of the measurement/study visit (until the end)
- Intake of alcohol:
  - Limit to two standard units on evenings before the measurement/study visit
  - Refrain on the days of the measurement/study visit (until the end)
- Intake of recreative drugs or self-medication (including cannabis oil):
  - Refrain on day before and the day of each measurement/study visit (until the end)
- Intake of benzodiazepine medications:
  - Exclusion or rescheduling when it is not possible to refrain day before and the day of each measurement/study visit (until the end)
  - Exception: low dose of benzodiazepine medication with intake in the evening, that functions as sleep medication
- Intake of corticosteroid medications:
  - (Temporary) exclusion, when exclusion criteria are no longer being met (see table S3)
- Smoking cigarettes, cigars or e-cigarettes:
  - Daily smoker: agreement on smoking schedule during measurements/study visits
  - Occasional smoker: refrain on the days of the measurements/study visit
- Instructions for collection of saliva, for each 60-minute period before sampling:
  - No intake of food
  - No intake of drinks, other than water
  - No smoking
  - No brushing teeth or flossing
  - No physical exercise

S7. Dexamethasone suppression test

Rationale

The dexamethasone suppression test (DST) is performed by the participant at home and involved sampling of saliva cortisol on two mornings directly after waking up at waking time, 15, 30, 45, 60 minutes and 8 hours after waking time. Dexamethasone (0.5mg) was orally administered at 11pm on the evening before the second morning assessment. In the current study, the results of the DST are one of the primary outcome measures to investigate dynamics of the HPA axis. Here, were are interested in group-level differences (PTSD versus control group), but also individual differences within the PTSD group (i.e. associations with other outcomes / predictive value). Optimisation of procedures to ensure reliability were therefore considered to be of vital importance. In the light of concentration and memory complaints as well as sleep disturbances for many people with PTSD, participants receive in-person instructions during a study visit and several documents with supporting information. Procedures can also be tailored to the individual in order to facilitate successful completion of the test.

Materials

Overview of instructions

This document contains contact details of the research team, an overview of supporting documents, a two-page overview of the procedure – from making a plan with the researcher until the end of the test – and a large section of frequently asked questions. The frequently asked questions concern dexamethasone, test procedures, and what to do in case of a mistake or deviation from the planned procedure.

Personal plan

The template of the personal plan contains sections to note down agreements on the following topics:

- Sleep disturbances: does the participant experience sleep disturbances? More specifically, do they wake up in the night and then not fall asleep again? The test should be started when the participant wakes up in the morning. If they wake up in the night, they should go back to sleep. However, if they wake up in the early morning (e.g. at 5.00 AM), plan to go back to sleep, but cannot fall back to sleep again, then they would have missed the moment in which they should have started the test. This is explained to the participants, and it is discussed when the participant should (or should not) start with the test in the context of their personal situation.
- Days of the procedure: which days would suit for the participant to conduct the procedure? It is instructed that these days should be similar in terms of activity level, and similar in terms of circumstances during sleep in the night before the test (e.g. location, with or without partner, amount of daylight in the morning, use of sleep medication the night before). The days do not have to be successive, but preferably they should be within the same week. Intake of dexamethasone should be at least three days before the next study visit in order to prevent carry-over effects.
- Use of an alarm clock: does the participant usually set an alarm clock in the morning? At which time? During the study, it is recommended to set an alarm clock for both days,

preferably at the same time, at a time that prevents natural waking before the alarm clock. This will promote similarity between the test days and accuracy of the procedures, because the timing of each sample will be known in advance (and reminders can be prepared on the day before).

- Use of reminders: would it work for the participants to set alarms (on their mobile phone) not only for waking up, but also for each time that a saliva sample should be taken? Do they want to use any other system for reminders?
- Other suggestions: does the participant have any suggestions of their own regarding accurate performance of the test?
- Smoking: if the participants smokes cigarettes/cigars/e-cigarettes on daily basis, they should ensure that their smoking pattern is equal across both days, so that the days can be compared fairly. They should never smoke in the 60-minute period prior to a saliva sample, otherwise they can determine their own pattern of smoking for both days (preferably at their usual frequency).

Logbook

The logbook contains several sections with questions, checklists, and brief information on factors that are important during the test:

- Smoking schedule (if applicable): first a section on agreements that were made, after that a log of the actual smoking frequency on both days
- Instructions for the logbook table regarding collection of saliva samples
- Checklist for preparations (day 1):
    - All materials are next to the bed
    - Time can be read directly after waking up (preferably on a device that is connected to internet)
    - Alarm clock and reminders have been set
    - Pill reminder app is prepared and immediately available
    - Participant is wearing the fitbit device
- Checklist for the day of testing (day 1):
    - Agreements related to use of alcohol, caffeine, recreative drugs, benzodiazepine medication, and smoking
    - Instructions for quality insurance of saliva samples
- Medication log (day 1): report of the (prescribed) medications that are taken on the day of testing, including sleep medications on the evening before the test
- Logbook form (day 1):
    - Reminder to first take the first saliva sample directly after waking
    - General information: date, activity level of the day (quiet or busy day, with room for comments)
    - Information about waking: time of the alarm clock, time of waking (note: change reminders if you did not wake up at the time of the alarm), reliability of the waking time if not waking at the time of the alarm
    - Information about sleep and nightmares: did you have a nightmare just before waking in the morning, if so, how much stress did you experience (score 1-10) and how much physiological stress (e.g. sweating, quick breathing, palpitations) did you

experience (score 1-10), how would you rate your sleep quality (score 1-10, with room for comments) and is this worse / similar / better than you usually slept in the past month.
    - Logbook table regarding collection of saliva samples
    - Storage of collected saliva sampling in the fridge
    - Comments
- Intake of dexamethasone:
    - Reminder to take on the evening prior to day 2 of the test at 11 PM
    - Checklist item: intake of dexamethasone completed
    - Date and time of intake
- Checklist for preparations (day 2): same as for day 1
- Checklist for the day of testing (day 2): same as for day 1
- Medication log (day 2): same as for day 1
- Logbook form (day 2): same as for day 1

Background information

More detailed explanation of the rationale behind the dexamethasone suppression test, the HPA axis, and the role of dexamethasone intake during the test.

Pill reminder app

The "Pill reminder app and Med tracker" (developed by Android developer "Sergio Licea") is available for Android and Apple devices. It is available with free download and no registration is needed. Participants are recommended to use this app as digital logbook for the timing of saliva sampling and intake of dexamethasone. The reminder features are not used. Participants receive a document with step-to-step instructions about the use of this app.

Procedure

Study visit A1

- Introduction: the participant is informed that it is very important that the test is conducted as accurately as possible. To help them achieve this, we make a personal plan together.
- Brief background information: the participant is briefly informed on the rationale of the test, so that they can understand better what is expected of them and how they can contribute with their own suggestions. It is emphasised that the two days of the test are being compared to test the biological stress system, therefore it is important that they are similar in many ways. Participants are also informed about the mechanism of dexamethasone and that it is not expected that they will experience any side effects. If they would like more information about this test, they can read the document "background information" (optional).
- Brief introduction of all materials: see section "materials" above.
- Reviewing of "overview of instructions" document: it is emphasised to the participant that adherence to the strict time schedules for saliva sampling is very important for the reliability of results from this test. It is a simple test, but there many things to take into consideration with its execution, so in its totality it can be challenging. All documents are meant to support the test procedure, everything that is discussed during the study visit is also outlined in the

documents. The section frequently asked questions can be addressed if they have a questions when they later return home.

- Memory and/or concentration problems are normalised to the participant in the context of PTSD, and it is asked whether they recognise these complaints. The researcher then takes this into account when discussing the test procedure. It is encouraged that the participant brings up their own suggestions for successful completion of the test procedures.
- Completing the template of the “personal plan” document.
- Reviewing of “logbook” document: all sections of the logbook are reviewed with the participant. Completion of the logbook table regarding collection of saliva samples is practised using different examples, in which there are small deviations from the planned procedure. These are the same examples that are outlined within the logbook at the section about instructions for the logbook table regarding collection of saliva samples, so that they can be reconsulted by the participant at home.
- Important comments by the researcher:
    - Take the time to prepare for the procedure on the day before; read the checklists in the logbook and make sure that all materials are immediately visible and readily available after waking up.
    - Do not ‘snooze’ on the test days, the results will not be reliable. It is best to get out of your bed, or do an activity (e.g. reading) to prevent falling back to sleep. After collection of the morning saliva samples, napping is allowed.
    - After waking, immediately take note of your waking time and start with the first saliva sample.
    - If you are using the Pill reminder app, register each collection of a saliva sample at the start of the saliva sampling collection procedure.
    - If you deviate from the planned time schedule for collection of saliva samples; if you are some minutes too late, immediately start collecting the sample and accurately report the actual time of collection in the logbook table. The next saliva sample should collected at the originally planned timing (i.e. the interval will be shorter). In case of larger deviations from the planned procedure, please look at frequently asked questions
    - If you have any questions when you are at home, or if there is a deviation from the planned procedure, you can review the section frequently asked questions. It will inform what to do. Please contact the research team if that is requested or when your question remains.
    - With this procedure, there are many things to take into account, this can be a challenge. Do not worry if you forget something, or when there are small, accidental deviations from the planned procedure. Just report as accurately as possible any deviations, so that we can evaluate the quality of the test results. Sometimes, we may be able to correct for deviations. If the procedure fails, it is also possible to try again later – we can discuss this when it happens.
- Instructions for Fitbit device: the device should be worn on the nights before each test day, on the non-dominant hand, tightly wrapped around the wrist. The researcher and participant discuss and demonstrate the placement around the wrist
- Preparation of the Pill reminder app: the app is installed on the participant’s mobile phone and prepared for use by adding “saliva sample” and “dexamethasone” under medications,

with frequency 'as needed'. It is explained to the participant how they can add an 'as needed' medication intake, to create an online log with time stamps of sampling and dexamethasone intake. The convenience of the app is that the time stamp is automatically saved in an accurate manner. Participants can use the app as logbook, and later copy the exact times to the paper logbook.

- Collection of test saliva sample: the procedure of collecting a saliva sampling is explained and then practiced by the participant, including the use of the Pill reminder app (if applicable).
- Does the participant have any questions?
- Control questions, for which the participant has access to all documents, to check their understanding and give them confidence with regard to the test procedure:
    - Where can you find the agreed plans for the test?
        - Personal plan
    - What are the agreements for the test procedure (e.g. regarding intake of alcohol), where can you find it?
        - Information and checklist in logbook
        - Frequently asked questions
    - On the day of the test, what do you do (in the correct order of events)?
        - Look immediately at the clock and remember waking time
        - Collect the first saliva sample; this is readily available next to the bed
        - Use the pill reminder app (or fill in the paper logbook)
        - Then continue with the questions in the paper logbook, next saliva samples is scheduled for 15 minutes after waking time
    - Can you snooze on the morning of the test procedure
        - No
    - When will you get the dexamethasone (in case it is scheduled for delivery at their home address)?
    - When do you need to take the dexamethasone?
        - On the evening prior to test day 2, at 11 pm; the participant should be able to translate this to their own planning
    - What do you do if you have a question about the test when you get home?
        - Look at section frequently asked questions, then contact research team

Study visit A2

The participant returns the logbook, fitbit device and collected saliva samples. If the participant used the Pill reminder app, the researcher takes photographs of the online logs from the test days in the app. The researcher discusses with the participant any comments, mistakes or deviations from the plan regarding the test procedures. Furthermore, the completeness and accuracy of the logbook is checked and supplemented by the researcher in case any information is missing. When researcher evaluates the quality of the test results as being insufficient, they discuss with the participant if they are willing to repeat the procedure, and receive extra reimbursement for this additional time investment.

S8. Socially evaluated cold-pressor test

Shortly before the procedure, the researcher introduces that "the expert" will take over and leaves the test room. The researcher who is referred to as "the expert" is female and unknown to the participants, and she functions as "stressor". During the entire procedure, she maintains a neutral tone, formal attitude, and refrains from any form of verbal or non-verbal social encouragement. The stressor enters the room to conduct the stress procedure. After instructions from the stressor, the onset of the procedure is marked by the moment that the hand of the participant is submerged in cold water (1.5-2.5 °C). At this point, the duration of the procedure is unknown to the participant. The procedure finishes after three minutes, or when the participant declares they no longer endure the procedure. The stressor then indicates the end of the procedure and leaves the room.

S9. Fear conditioning and extinction task: additional information and experimental setup

Task implementation

The task was developed and runs in Unity (version 2019.2.6f1). Navigation within the environment and providing shock expectancy ratings is achieved by using a joystick. In the task, stimuli are presented for 5.0 seconds. During the first 4.5 seconds of stimulus presentation, the participant can provide their shock expectancy rating. When they first move the joystick upwards or downwards, number 3 (i.e. is the middle number of the rating scale) is selected. They can move the joystick again to select the number of their choice. After 4.5 seconds, the rating is automatically confirmed. During the acquisition phase, shocks are delivered in some trials during the last 200ms of stimulus presentation. A fixation cross is with random duration of between 5.5 seconds and 8.5 seconds is presented at the start and end of each block and in-between trials.

Task configuration

Task settings of the fear conditioning and extinction task (e.g. stimulus order) are individually configured for counterbalancing and (pseudo) randomization of task features. Details on the task configuration are provided in table S4. Individual task configuration files corresponding to randomization numbers were created with a script in Python with the use of a random seed to ensure reproducibility.

Experimental setup

Shock electrodes are attached to the middle- and ring finger of the hand that is also used for joystick navigation. Electrodes to measure skin conductance response (SCR) are attached to the hand on the middle- and ring finger, and the pulse wave sensor is attached to the index finger of the other hand. All electrodes and the pulse wave sensor are attached with tape to the distal phalanges of the participant. Conductive gel is used with application of electrodes. Pupil dilation of the left eye is measured with a sampling rate of 1000Hz after a short five-point calibration procedure to verify successful recognition of the pupil. The abovementioned physiological responses are monitored during all phases of the fear conditioning and extinction task using a BrainAmp ExG MR amplifier. During the MRI session, respiration is additionally measured using a respiratory device placed under a belt on the diaphragm.

Shock calibration procedure

Participant instructions

The participants receive the following instructions regarding the individual calibration of the shock intensity (as translated from Dutch):

"We will now determine the settings of the shocker device. The shocks are meant to be really uncomfortable, but not too painful. We will determine the right settings for the shock for you. Starting with a low shock intensity, you will be asked on a scale from 1 to 5 how uncomfortable and painful you experienced the shock exposure. Here, 1 reflects that you barely felt the exposure, and 5 reflects that it was too painful. We will test with five exposures, after which the final level will be determined. I would like to emphasise that the current study investigates stress, therefore it is really important that the shock is truly uncomfortable, otherwise the research will fail. From experience we know that

people will usually get used to receiving the shock. We would like to find an intensity level for which this will not happen."

Table S4. Counterbalancing and (pseudo) randomization in individual task configuration settings

Task setting	Details
Virtual environment	Determines which virtual environment is loaded in which study session. This setting is counterbalanced for by including it in the counterbalancing scheme, see section "counterbalancing"
Joystick movement direction	Determines direction of joystick movement of scoring. In one setting, pushing the joystick away will result to a lower score on the rating scale and pulling towards will result in a higher score on the rating scale; in the other setting this is implemented vice versa. This setting remains constant across both study sessions. In a randomized order, for each block of six rows within the counterbalancing scheme, three rows have the one setting and the other three rows have the other setting.
Scheme for conditioned stimuli (CSs)	Determines which shape corresponds to which type of CS. Three different shapes provide six possible shape-CS combinations. In random order, for each block of six rows within the counterbalancing scheme, each shape-CS combination is included once.
Scheme for contexts	Determines which house corresponds to which type of context. Three different houses provide six possible house-context combinations. In random order, for each block of six rows within the counterbalancing scheme, each shape-CS combination is included once. With regard to both study sessions, task settings ensure that the location of all contexts are different, even though houses are at the same location within the environment.
Stimulus sequence fear acquisition	Determines the sequence of stimuli in the first phase of the task. Each task block contains four stimuli of each type, that is twelve stimuli in total, for which the order was pseudo randomized with the criterion of no more than three of the same consecutive stimuli. CS-type sequences were equal between study sessions.
Shock trials during fear acquisition	Determines which trials during the acquisition phase include delivery of shock. Each task block contains four stimuli of each type. Two stimulus types are sometimes followed by shock according to a 50% reinforcement rate, this translates to two trials for those stimulus types that were randomly selected. Shock trial sequences were equal between study sessions.
Stimulus sequence fear extinction	Determines the sequence of stimuli in the second phase of the task. Each task block contains four stimuli of each type that is included in this phase, that is eight stimuli in total, for which the order was pseudo randomized with the criterion of no more than three of the same consecutive stimuli. CS-type sequences were equal between study sessions.
Stimulus sequence recall	Determines the sequence of stimuli in the third phase of the task. Each context has two blocks, and each block contains two stimuli of each type. The order of three stimuli are counterbalanced for by including it in the counterbalancing scheme, see section "counterbalancing". This stimulus order is applied to the first three stimuli in each context, otherwise the sequence of stimuli was random. CS-type sequences were equal between study sessions.

Calibration procedure

The intensity level of the shock exposure is individually calibrated to be uncomfortable, but not too painful, according to a standardised procedure that involves exactly five shock exposures. Shock intensities correspond to ten intensity levels (1-10 of 0-40 V at 0-80 mA. The initial procedure is outlined in Figure S1.

Shock level:	Participant rating (change for next exposure)					Comments
**Start level: 2**	1 (+2)	2 (+1)	3 (=)	4 (=)	5 (-1)	
	1 (+2)	2 (+1)	3 (+1)	4 (=)	5 (-1)	
	1 (+3)	2 (+2)	3 (+1)	4 (=)	5 (-1)	
	1 (+3)	2 (+2)	3 (+1)	4 (=)	5 (-1)	If rating 4, for 2nd time: "Shall we try one level higher? If it is too painful, we will go back to this level"
	1 (+3)	2 (+2)	3 (+1)	4 (=)	5 (-1)	"Would this be an intensity level that you would not get used to?"
**End level:**						

Figure S1. Procedure for individual calibration of the shock intensity level. The first exposure has shock intensity level 2. After each exposure, the participant is asked to provide a subject rating on a scale from 1 to 5 regarding how uncomfortable and painful they experienced the shock exposure. Based on the rating of the participant, the shock level may be adjusted (middle column). Throughout the procedure, the participant is encouraged to try higher shock exposure levels when their shock level and rating have remained constant for two trials (right column). The final level is found in exactly five exposures.

After this initial calibration procedure, the researcher asks (as translated from Dutch):

> *"How much would you dread receiving another shock (with the finally determined intensity level), on a scale from 1 (not at all) to 10 (very much)?"*

If the participant rating is 5 or lower, the researcher instructs (as translated from Dutch):

> *"From experience we know that people will quickly get used to the shock, and then don't mind it so much anymore. For this research, it is very important that you now really would dread receiving a shock. Your rating is very low for the research, here there is a risk that the measurements will fail. I would like to suggest to increase the intensity with one level, if that is OK with you?"*

If the participant consents, the shock intensity is then increased with one level. The researcher asks again how much the participant would dread the shock now that the intensity level has increased.

Habituation

After completion of the acquisition phase, the researcher asks another question in order to ascertain and act on habituation to the shock (as translated from Dutch):

> *"How much would you now dread receiving another shock (with the finally determined intensity level), on a scale from 1 (not at all) to 10 (very much)?"*

If the participant's subjective rating on the above question 4 or lower, the researcher instructs (as translated from Dutch):

> *"Based on your rating, it appears that you have gotten used to the intensity of the shock, because you do not dread so much anymore to receive them. This is a problem for our research. Therefore, I suggest to increase the intensity with one level for the rest of the measurements today and tomorrow."*

If the participant's subjective rating on the above question is 5 or higher, the researcher instructs (as translated from Dutch):

> *"Based on your rating, it appears that the intensity of the shock is still at the appropriate level. You still dread to receive another shock. For the rest of the measurements today and tomorrow we will use this same intensity level."*

Practice tasks

Familiarization task

The first practice tasks familiarizes the participant with joystick navigation and with the VE of the main task; this is a nature or city environment, depending on the individual task configuration. The participant completes the practice task in the VE corresponding to the VE of the fear conditioning and extinction task for the respective session. They are instructed to find objects that are part of the environment, e.g. a tree or benches. A goal object is instructed by displaying the word in the upper right corner of the screen. After the participant navigates close to the object, the word changes to a check mark, after which a new goal is displayed. The researcher gives additional guiding instructions during the practice task when needed. In total, this practice task consists of eight goal objects in different corners of the VE.

Scoring practice task

The second practice tasks is to practice providing ratings with the joystick during the task, and to get familiar with the order of events in the main task. Participants are explicitly informed that this task is for practice; stimuli are not related to the main task and neither to the goal of the study. The VE of this practice task is different: a pink-coloured plane, without any objects, except the "instruction booth" and a simple blue booth. Timings of stimulus presentation and subject ratings are the same as in the fear conditioning and extinction task. Participants are instructed to alternate between the navigation booth and the blue booth. Within the instruction booth, they can give any rating on a scale from 1 to 5. In the blue booth, a block with pictures of different foods is presented (e.g. tomato, ice cream, bread, etc.). After each food item, they rate the healthiness of the presented food item on a scale from 1 ("unhealthy") to 5 ("healthy"). This practice task consists of four blocks of five food stimuli. The researcher gives additional guiding instructions during the practice task when needed. When the participant is not skilled enough yet at the end of the practice task with regard to giving ratings with the joystick, (part of) the practice task is repeated to ensure a sufficient amount of practice.

S10. MRI data acquisition

Brain imaging data are acquired with a 3T Skyra scanner (Siemens Healthineers, Erlangen, Germany) using a 32-channel head coil. Structural imaging includes a T1-MPRAGE-sequence (TR = 2300 ms, TE = 3.03 ms, flip angle = 8° , FOV = 256 mm, voxel size = 1.0 x 1.0 x 1.0 mm) and two diffusion-weighted sequences (TR = 3000 ms, TE = 93 ms, flip angle = 90°, FOV = 212 mm, voxel size = 2.0 x 2.0 x 2.0 mm, 5xb=0 + 40xb=1000 + 40xb=2000, MB acceleration factor = 3, phase-encoding direction = AP; and TR = 2930 ms, TE = 89.60 ms, flip angle = 90°, FOV = 212 mm, voxel size = 2.0 x 2.0 x 2.0 mm, 7xb=0, MB acceleration factor = 3, phase-encoding direction = AP). During the fear extinction and emotion processing tasks, blood oxygen-level dependent (BOLD) fMRI images are acquired with multiband gradient-echo echo planar imaging (EPI) sequences (TR = 1500 ms, TE = 33.4 ms, flip angle = 75°, FOV = 213 mm, voxel size = 2.0 x 2.0 x 2.0 mm, 68 slices, MB acceleration factor = 4, phase-encoding direction = AP). Resting-state images are acquired with a different EPI sequence (TR = 945 ms, TE = 28.00 ms, flip angle = 60°, FOV = 213 mm, voxel size = 2.0 x 2.0 x 2.0 mm, 66 slices, MB acceleration factor = 6, phase-encoding direction = AP). Three field map scans are acquired corresponding to each EPI sequence, twice (TR = 510 ms, TE 1 = 2.80 ms, TE 2 = 5.26 ms, flip angle = 60°, FOV = 213 mm, voxel size = 2.0 x 2.0 x 2.0 mm, 68 slices, phase-encoding direction = RL) and once (TR = 500 ms, TE 1 = 2.80, TE 2 = 5.26, flip angle = 60°, FOV = 213 mm, voxel size = 2.0 x 2.0 x 2.0 mm, 66 slices, phase-encoding direction = RL).

S11. Additional information on study procedures

First visit (A1)

During this visit, the participant completes the online SES questionnaire, the matrix reasoning task, and online questionnaires MACE and LEC-5. Here, the LEC-5 is being omitted if the questionnaire results are available from collaborating mental healthcare institutions or the screening visit. For the control group, the MACE is omitted as it has been completed during the screening visit.

Second visit (A2)

Prior to the visit, participants receive instructions for quality assurance of data (for details see Supplementary Materials S6). At the start of the visit, the participant first provides a subjective stress rating. The participant completes the following online questionnaires: BAI, DES-T, IDS-SR, MATE, PCL-5, PSQI, menstrual cycle questions and compliance questions. After providing another subjective stress rating, they are guided to another test room for the SECPT procedure. This test room is not used for other parts of this study. A baseline saliva sample and pre-measurements (stress rating, blood pressure, heart rate, PANAS) are collected by the researcher. Subsequently, the SECPT procedure is conducted. Immediately after the procedure, the researcher proceeds to conduct post-measurements (stress rating, blood pressure, heart rate, PANAS). Saliva samples and subjective stress ratings are collected at 15, 30, 45, 60, 75, and 90 minutes after the onset of the stress procedure. After the first saliva sample, the researcher and participant return to a general meeting room and the participant is instructed to wait until the next saliva samples are collected; they are not allowed to engage in any activities. At 45 minutes, the participant also receives a debriefing on the nature of the test and performance of the stressor, as well as positive feedback on their performance. Thereafter, participants are allowed to engage in other activities, such as reading or engaging with their phones. However, they are not allowed to engage with the researcher about their experiences until the last saliva sample.

MRI visit (B1.1 and B2.1)

All participants fill in the MRI screening form to ensure MRI safety, and women do a pregnancy test which must be negative before ingesting the study medication. They also complete the following online questionnaires: BAI, DES-T, IDS-SR, PCL-5, PSQI, and compliance questions. A practice session in the dummy MRI lab is conducted so that the participant is familiar with the procedures during the MRI session, and so that they feel more comfortable and less anxious. In the dummy scanner, all measurement devices (such as electrodes) are attached and multiple MRI sounds are played with the aim to establish a similar experience. After the MRI session, the participant receives a Fitbit device and is instructed to wear this during the upcoming night to measure their sleep.

Next-day visit (B1.2 and B2.2)

At the start of this visit, the participant returns the Fitbit device and completes the following online questionnaires: sleep quality of the previous night, menstrual cycle questions and compliance questions.

Additional patient data

Information that may be requested from the mental health institutions includes: LEC-5 questionnaire data; routine outcome measurement data, including (but not limited to) PCL-5 questionnaire data; diagnostic information (including psychiatric comorbidities); and information about type and duration of PTSD treatment. This information is retrieved to prevent unnecessary double questionnaire assessments (for LEC-5) and may further characterize (heterogeneity of) the study sample.

S12. Alternative analyses

Observational study

The mitigation plan for the observational study is to adapt statistical analyses in case extension of the data collection period will is no longer feasible and the planned sample size for the PTSD group (n=160) is not achieved. When the PTSD group consists of n<50 participants, we will only investigate objective (1) regarding HPA-axis in the PTSD group as compared to the control group. When the PTSD group consists of n≥50 participants, this will be considered sufficient to additionally investigate objective (2) about associations with ELA, as it will be similar to many previous clinical studies on this topic [24]. Objective (3) should only be investigated with n>120 participants. Alternatively, we will explore opportunities to combine data from research projects to investigate DNA methylation with sufficient power. Objective (4) will be explored only when n≥50 have completed the follow-up survey.

Pharmacological intervention study

The mitigation plan for the pharmacological intervention study is to adapt statistical analyses in case extension of the data collection period is no longer feasible and the planned sample size (n=80) is not achieved. Our pre-defined primary outcome variable for HPA-axis dysregulation is a grouping variable based on a median spilt on relative suppression of the CAR after administration of 0.5 mg dexamethasone, as measured with DST. This choice is theory based, based on its GR-mediated effect on HPA-axis negative feedback. Results from the observational may indicate, however, that this measure is insufficiently informative and/or other measures may be found to be more informative. For instance, when dexamethasone would cause almost full suppression of the CAR, this could lead to floor effects in morning cortisol levels, and consequently limited sensitivity to measure individual differences in GR sensitivity. In such cases, we will proceed our analyses with a different primary outcome measure for HPA-axis dysregulation. Statistical analysis plans will also be adapted when the final sample size of the PTSD group is below the planned sample size. The current analysis plans will be conducted with n≥50 participants. In case n<50 but n≥35 participants, reduced statistical power will be addressed by investigation of HPA-axis dysregulation as continuous variable.

S13. Psychophysiological analysis

Psychophysiological measures are preprocessed using in-house software. SCRs throughout the different phases of the fear conditioning and extinction task will be calculated by a trough-to-peak method, with a response onset window of 0.5-5.3 seconds after stimulus onset, and a response window of 10 seconds. For eye-tracking data, signal artefacts due to eye blinks will be removed using linear interpolation. Event-related PDRs are calculated by dividing averaged pupil dilation during the 3000-5000 ms (for fear acquisition phase: 3000-4800 ms) periods after frame onset by the averaged 1000 ms prior to onset.

S14. MRI analysis

Preprocessing and analysis of MRI data will require specialist software packages (fMRIprep, SPM, FSL, etc.). Firstly, fMRI data will be cleaned from noise artefacts and nuisance variables, and co-registered to T1-weighted structural images. Thereafter, the data will be transformed to standard space, defined by the template of the Montreal Neurological Institute (www.mni.mcgill.ca), by means of non-linear spatial transformation. This will be followed by spatial smoothing of the imaging data. For actual statistical evaluation, regressors derived from the experimental design of each task will be constructed by convolution with a canonical hemodynamic response function (HRF). First-level analyses of event-related activity will provide voxel-based brain activity measures for contrasts of interest per subject. Subsequently, averages of task-related neural activity across subjects and group differences therein will be estimated using higher-level general linear models that will include appropriate covariates. Subsequently, resulting statistical parametric maps will be superimposed upon structural images in order to localize brain activity. Regions of interest are those brain regions associated with fear expression and extinction. If appropriate, multiple comparisons corrections will be performed using random field theory-based corrections.

References

1. Sheehan D V., Lecrubier Y, Sheehan KH, et al (1998) The Mini-International Neuropsychiatric Interview (M.I.N.I.): The development and validation of a structured diagnostic psychiatric interview for DSM-IV and ICD-10. J Clin Psychiatry 59:22–33

2. Teicher MH, Parigger A (2015) The "Maltreatment and Abuse Chronology of Exposure" (MACE) scale for the retrospective assessment of abuse and neglect during development. PLoS One 10:1–37. https://doi.org/10.1371/journal.pone.0117423

3. Faul F, Erdfelder E, Lang A-G, Buchner A (2007) G*Power 3: A flexible statistical power analysis program for the social, behavioral, and biomedical sciences. Behav Res Methods 39:175–191

4. Bunea IM, Szentágotai-Tătar A, Miu AC (2017) Early-life adversity and cortisol response to social stress: A meta-analysis. Transl Psychiatry 7:1274. https://doi.org/10.1038/s41398-017-0032-3

5. Yehuda R, Flory JD, Bierer LM, et al (2015) Lower methylation of glucocorticoid receptor gene promoter 1F in peripheral blood of veterans with Posttraumatic Stress Disorder. Biol Psychiatry 77:356–364. https://doi.org/10.1016/j.biopsych.2014.02.006

6. Klengel T, Mehta D, Anacker C, et al (2013) Allele-specific FKBP5 DNA demethylation mediates gene-childhood trauma interactions. Nat Neurosci 16:33–41. https://doi.org/10.1038/nn.3275

7. Yehuda R, Golier JA, Halligan SL, et al (2004) The ACTH response to dexamethasone in PTSD. Am J Psychiatry 161:1397–1403. https://doi.org/10.1176/appi.ajp.161.8.1397

8. de Quervain DJ-F, Bentz D, Michael T, et al (2011) Glucocorticoids enhance extinction-based psychotherapy. Proc Natl Acad Sci 108:6621–6625. https://doi.org/10.1073/pnas.1018214108

9. Uschner D, Schindler D, Hilgers RD, Heussen N (2018) randomizeR: An R package for the assessment and implementation of randomization in clinical trials. J Stat Softw 85:. https://doi.org/10.18637/jss.v085.i08

10. Boeschoten MA, Van der Aa N, Bakker A, et al (2018) Development and evaluation of the Dutch Clinician-Administered PTSD Scale for DSM-5 (CAPS-5). Eur J Psychotraumatol 9:1546085. https://doi.org/10.1080/20008198.2018.1546085

11. Christy A. Blevins, Weathers FW, Davis MT, et al (2015) The Posttraumatic Stress Disorder Checklist for DSM-5 (PCL-5): Development and initial psychometric evaluation. J Trauma Stress 28:489–498. https://doi.org/10.1002/jts.22059

12. Boeschoten MA, Bakker A, Jongedijk RA, Olff M (2014) PTSD Checklist for DSM-5 – Nederlandstalige versie. Stichting Centrum '45, Arq Psychotrauma Expert Groep, Diemen

13. Rush AJ, Carmody T, Reimitz P-E (2000) The Inventory of Depressive Symptomatology (IDS): Clinician (IDS-C) and Self-Report (IDS-SR) ratings of depressive symptoms. Int J Methods Psychiatr Res 9:45–59. https://doi.org/10.1002/mpr.79

14. Beck, Brown, Epstein, Steer (1988) An inventory for measuring clinical anxiety: Psychometric properties. Journal of Consulting and. Clin Psychol 56:893–897

15. Waller NG, Putnam FW, Carlson EB (1996) Types of dissociation and dissociative types: A taxometric analysis of dissociative experiences. Psychol Methods 1:300–321. https://doi.org/10.1037/1082-989X.1.3.300

16. Schippers GM, Broekman TG, Buchholz A (2011) MATE 2.1. Handleiding en protocol.

17. Carpenter JS, Andrykowski MA (1998) Psychometric evaluation of the Pittsburgh Sleep Quality

Index. J Psychosom Res 45:5–13. https://doi.org/10.1016/S0022-3999(97)00298-5

18. Buysse DJ, Reynolds CF, Monk TH, et al (1989) The Pittsburgh Sleep Quality Index: A new instrument for psychiatric Ppractice and research. Psychiatry Res 28:193–213

19. Haghayegh S, Khoshnevis S, Smolensky MH, et al (2020) Performance assessment of new-generation Fitbit technology in deriving sleep parameters and stages. Chronobiol Int 37:47–59. https://doi.org/10.1080/07420528.2019.1682006

20. Watson D, Clark LA, Tellegen A (1988) Development and validation of brief measures of positive and negative affect: The PANAS scales. J Pers Soc Psychol 54:1063–1070

21. Weathers FW, Blake DD, Schnurr PP, et al (2013) The life events checklist for DSM-5 (LEC-5)

22. Adler NE, Epel ES, Castellazzo G, Ickovics JR (2000) Relationship of subjective and objective social status with psychological and physiological functioning: Preliminary data in healthy white women. Heal Psychol 19:586–592. https://doi.org/10.1037/0278-6133.19.6.586

23. Arthur W, Day D V (1994) Development of a short form for Raven Advanced Progressive Matrices test. Educ Psychol Meas 54:394–403

24. Houtepen LC, Vinkers CH, Carrillo-Roa T, et al (2016) Genome-wide DNA methylation levels and altered cortisol stress reactivity following childhood trauma in humans. Nat Commun 7:10967. https://doi.org/10.1038/ncomms10967
